# The repetitive landscape of the 5100 Mbp barley genome

**DOI:** 10.1186/s13100-017-0102-3

**Published:** 2017-12-20

**Authors:** Thomas Wicker, Alan H. Schulman, Jaakko Tanskanen, Manuel Spannagl, Sven Twardziok, Martin Mascher, Nathan M. Springer, Qing Li, Robbie Waugh, Chengdao Li, Guoping Zhang, Nils Stein, Klaus F. X. Mayer, Heidrun Gundlach

**Affiliations:** 1 Department of Plant and Microbial Biology, University of Zurich, Zollikerstrasse 107, CH-8008 Zurich, Switzerland; 2 Institute of Biotechnology and Viikki Plant Science Centre, University of Helsinki, Helsinki, Finland; 3 Green Technology, Natural Resources Institute Finland (Luke), Helsinki, Finland; 4 PGSB - Plant Genome and Systems Biology, Helmholtz Center Munich - German Research Center for Environmental Health, Neuherberg, Germany; 5 Leibniz Institute of Plant Genetics and Crop Plant Research (IPK), Seeland, Germany; 6 German Centre for Integrative Biodiversity Research (iDiv) Halle-Jena-Leipzig, Leipzig, Germany; 7 Department of Plant and Microbial Biology, University of Minnesota, 1479 Gortner Avenue, Saint Paul, MN 55108 USA; 8 Present address: National Key Laboratory of Crop Genetic Improvement, Huazhong Agricultural University, Wuhan, 430070 China; 9 The James Hutton Institute, Dundee, UK; 10 School of Life Sciences, University of Dundee, Dundee, UK; 11 Western Barley Genetics Alliance/the State Agricultural Biotechnology Centre, School of Veterinary and Life Sciences, Murdoch University, Murdoch, WA6150 Australia; 12 Department of Primary Industry and Regional Development, Government of Western Australia, South Perth, WA6155 Australia; 13 College of Agriculture and Biotechnology, Wuhan, ZU China; 14 TUM School of Life Sciences Weihenstephan, Technical University of Munich, Freising, Germany

## Abstract

**Background:**

While transposable elements (TEs) comprise the bulk of plant genomic DNA, how they contribute to genome structure and organization is still poorly understood. Especially in large genomes where TEs make the majority of genomic DNA, it is still unclear whether TEs target specific chromosomal regions or whether they simply accumulate where they are best tolerated.

**Results:**

Here, we present an analysis of the repetitive fraction of the 5100 Mb barley genome, the largest angiosperm genome to have a near-complete sequence assembly. Genes make only about 2% of the genome, while over 80% is derived from TEs. The TE fraction is composed of at least 350 different families. However, 50% of the genome is comprised of only 15 high-copy TE families, while all other TE families are present in moderate or low copy numbers. We found that the barley genome is highly compartmentalized with different types of TEs occupying different chromosomal “niches”, such as distal, interstitial, or proximal regions of chromosome arms. Furthermore, gene space represents its own distinct genomic compartment that is enriched in small non-autonomous DNA transposons, suggesting that these TEs specifically target promoters and downstream regions. Furthermore, their presence in gene promoters is associated with decreased methylation levels.

**Conclusions:**

Our data show that TEs are major determinants of overall chromosome structure. We hypothesize that many of the the various chromosomal distribution patterns are the result of TE families targeting specific niches, rather than them accumulating where they have the least deleterious effects.

**Electronic supplementary material:**

The online version of this article (10.1186/s13100-017-0102-3) contains supplementary material, which is available to authorized users.

## Background

The genomes of higher plants vary dramatically in size, ranging from the 63.6 Mb of *Genlisea aurea* [1] to the almost 500-fold larger genomes of *Fritillaria* species [2, 3]. Among the angiosperms that have been examined, the mean monoploid genome size is 4723 Mb (Additional file 1: Figure S1), closely matching the 5100 Mb barley genome in size [4]. However, all diploid plant genomes sequenced so far contain approximately 20,000 to 35,000 genes. The differences per monoploid genome size are due to varying amounts of sequence derived from transposable elements (TEs). TEs are generally divided into retrotransposons (Class I) and DNA transposons (Class II, [5], which are further subdivided into orders and superfamilies. TEs can be viewed as genomic parasites. Autonomous (“master copy”) TEs encode the genes that enable them to replicate and move around in the genome (e.g.*,* reverse transcriptase, integrase, or transposase). In addition, they often give rise to large populations of deletion derivatives (non-autonomous TEs) that lack some or all coding capacity [5]. For non-autonomous elements to be replicated or transposed, they usually must have conserved sequence motifs that can be recognized by the mobilizing protein(s) encoded by the autonomous elements to allow their transposition.

**Fig. 1:**
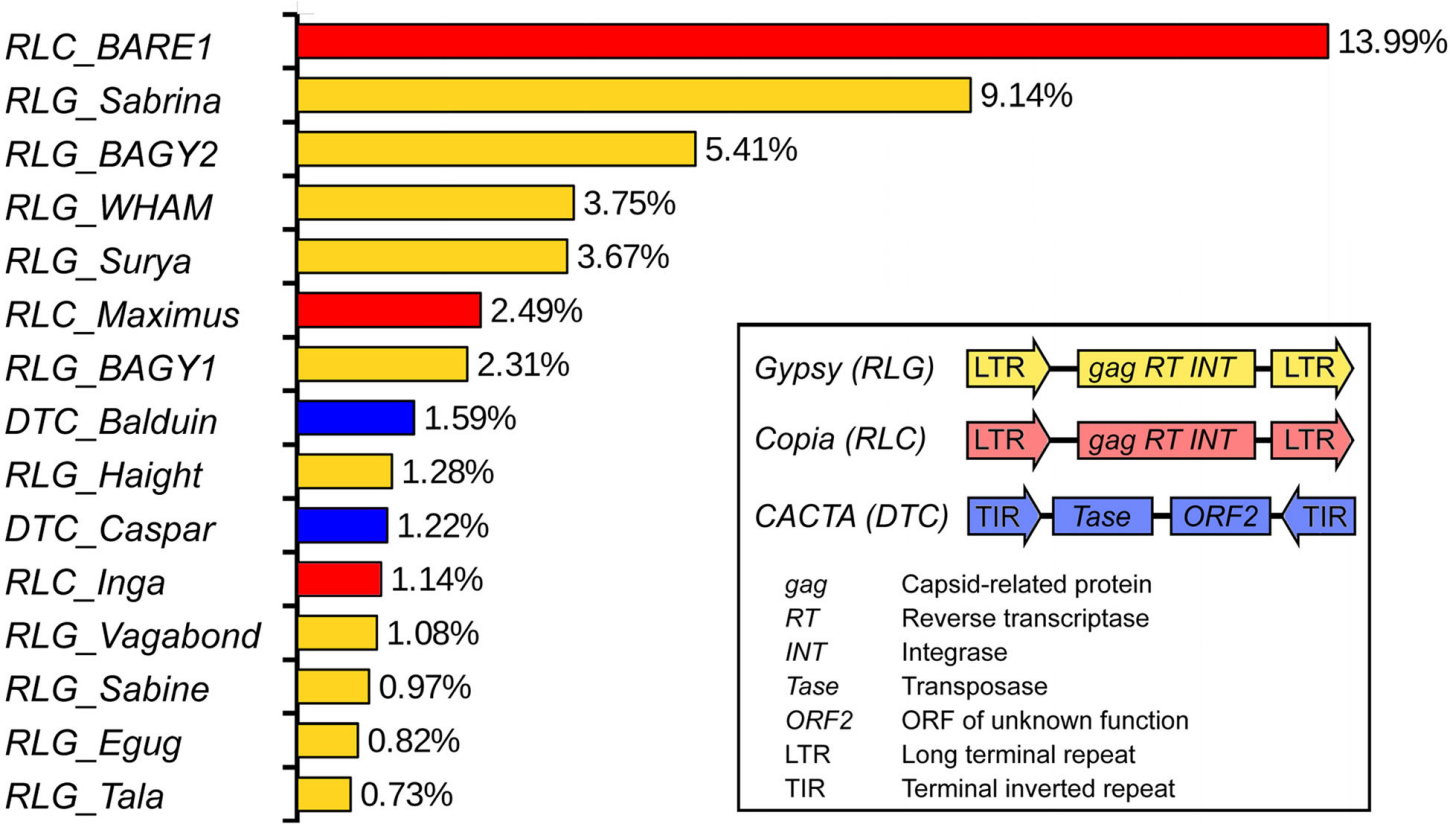
Contribution to total genome sequence of the top 15 TE families in the barley genome. Note that 10 of the top 15 TE families belong to the *Gypsy* superfamily (prefix “*RLG_*”). The *Copia* superfamily is represented with 3 families (prefix “*RLC_*”). The only Class 2 superfamily represented in the top 15 are *CACTA* elements (prefix “*DTC_*”). The inset shows the schematic sequence organization of these three superfamilies

The TE landscapes of all plant genomes sequenced so far are dominated by a small number of high-copy families [6–9]. In all cases, the TE fractions are composed primarily of long terminal repeat (LTR) retrotransposons [5]. The LTR retrotransposons described so far in plants belong either to the *Gypsy* or the *Copia* superfamily, two ancient lineages that differ in the order of the encoded genes for reverse transcriptase and integrase [5], Fig. 1). In plants with large genomes such as wheat, barley or maize, LTR retrotransposons are known to contribute at least 50% of the total TE content [6–9]. Especially the retrotransposon fraction of the 2300 Mb maize genome has been analyzed in great detail [10, 11]. Baucom et al. [10] identified over 400 families of retrotransposons in the maize genome. Generally, *Gypsy* elements were found to be enriched in pericentromeric regions, while *Copia* elements accumulated in distal chromosomal regions. Interestingly, high-copy families tend to cluster in gene-poor regions while low-copy elements were found often near genes, which was interpreted as a mechanism to increase the chances of less abundant elements to be activated and replicated [10]. Furthermore, different types of retrotransposons were found enriched in different chromosomal regions. For example, the “Sireviruses” [12], a large clade of *Copia* elements were found to be enriched in distal chromosomal regions [11].

DNA transposons typically contribute less to the total genomic DNA, but they show an extreme diversity. The largest fraction of DNA transposons is usually contributed by *CACTA* transposons, due to their large size and high copy numbers [4, 9, 13, 14]. Additionally, all grass genomes described so far are populated by tens of thousands of small non-autonomous DNA transposons. These small TEs (often referred to as miniature inverted-repeat transposable elements, or MITEs [15, 16] are preferably located near genes, suggesting an influence on the evolution of genes [17–19].

The extreme abundance and complexity of the TE fraction in large genomes has often resulted in highly fragmented genome assemblies that have hampered detailed analyses of their TE landscapes. The production of a high quality, nearly complete barley genome sequence [4] provided the opportunity to analyze in detail the abundance, distribution and target site preference of TE groups and individual TE families. As the barley genome is so far the largest plant genome sequenced and assembled to this level, we were particularly interested in exploring what role TEs have played in shaping it.

## Results

Overall, 80% of the barley genome was classified as derived from TEs [4], but the actual percentage is probably higher because of families with highly diverse members, which may have escaped detection by homology searches against known TEs. We observed that the barley genome is dominated by only a few TE families, as previous studies have suggested [6, 8]: ten *Gypsy,* three *Copia*, and two *CACTA* families together comprise over 50% of the whole genome (Fig. 1). We estimated copy numbers of TE families by dividing the total number of annotated base pairs by the length of the reference (consensus) sequence for the respective TE**.** Especially with large elements such as retrotransposons, this is problematic, since many copies are fragmented by deletions or reduced to solo-LTRs through intra-element recombination. Furthermore, individual families are sometimes comprised of different subfamilies of varying size (see below). Copy number estimates based on consensus sequences therefore have to be taken with caution. Using this approach, we estimate that the top 10 TE families by abundance together represent approximately 230,000 individual copies (Table 1). As previously described [6–8, 20], the *Copia* family *RLC_BARE1* is the most abundant in terms of copy numbers (> 76,000) as well as absolute contribution to the genome (> 14%, Fig. 1, Table 1). The rest of the repetitive landscape is comprised of at least 350 TE families with moderate or low copy numbers.

**Table 1:** Copy number estimates of the most abundant Class 1 and Class 2 element families in the barley genome

TE family	Superfamily	Total kb^a^	Length^b^	Copy number^c^
*RLC_BARE1*	*Copia*	623,043	8630	72,195
*RLG_Sabrina*	*Gypsy*	407,047	8030	50,691
*RLG_BAGY2*	*Gypsy*	240,798	8630	27,902
*RLG_WHAM*	*Gypsy*	167,138	9450	17,687
*RLG_Surya*	*Gypsy*	163,300	14,470	11,285
*RLC_Maximus*	*Copia*	110,928	14,400	7703
*RLG_BAGY1*	*Gypsy*	102,843	14,400	7142
*DTC_Balduin*	*CACTA*	70,688	11,740	6021
*RLG_Haight*	*Gypsy*	57,185	13,080	4372
*DTC_Caspar*	CACTA	54,465	11,568	4708
Total		1,997,435		209,707
*DTT_Thalos*	*Mariner*	2865	163	17,574
*DTT_Pan*	*Mariner*	716	123	5822
*DTT_Athos*	*Mariner*	394	81	4868
*DTT_Icarus*	*Mariner*	555	117	4747
*DTT_Hades*	*Mariner*	392	108	3627
*DTT_SAF*	*Mariner*	177	85	2087
*DTT_Eos*	*Mariner*	506	326	1552
*DTT_Oleus*	*Mariner*	231	150	1540
*DTT_Pluto*	*Mariner*	328	274	1197
*DTT_Stolos*	*Mariner*	205	274	749
Total		6369		43,763
*DTH_Thorne*	*Harbinger*	716	273	2624
*DTH_Kerberos*	*Harbinger*	594	285	2086
*DTH_Xumet*	*Harbinger*	591	376	1571
*DTH_Rong*	*Harbinger*	1218	1227	993
*DTT_Marimom*	*Harbinger*	2024	2129	951
*DTH_Orpheus*	*Harbinger*	183	272	674
*DTH_Xenon*	*Harbinger*	203	312	650
*DTH_Xian*	*Harbinger*	650	1161	560
*DTH_Kong*	*Harbinger*	489	2119	231
*DTH_Tibone*	*Harbinger*	187	1037	180
*DTH_Zong*	*Harbinger*	278	2396	116
Total		7133		10,634

^a^Total kb annotated as respective family-specific

^b^Length of the reference TE that was used for annotation

^c^Copy number estimate based on total kb occupied by the TE family and length of its consensus sequence

In addition to the large *Gypsy*, *Copia*, and *CACTA* elements, which can range in size from roughly 2 kb to over 30 kb (deposited in TREP, see methods), the barley genome also contains approximately 54,000 small DNA transposons of the *Mariner* and *Harbinger* superfamily (Table 1). However, due to their small size, their contribution to genome size is negligible.

### The barley genome contains large populations of non-autonomous retrotransposons

To study gene content and coding capacity of TEs, we constructed consensus sequences of individual TE families using at least 3, but sometimes up to 100 copies. Although many individual TE copies might be degenerated, construction of consensus sequences usually leads to the emergence of intact ORFs which were used for prediction of hypothetical proteins. Because individual families sometimes diverged into different subfamilies, we also constructed consensus sequences for individual subfamilies. If a consensus sequence contained no intact ORFs, the respective TE family (or subfamily) was considered non-autonomous.

**Fig. 2:**
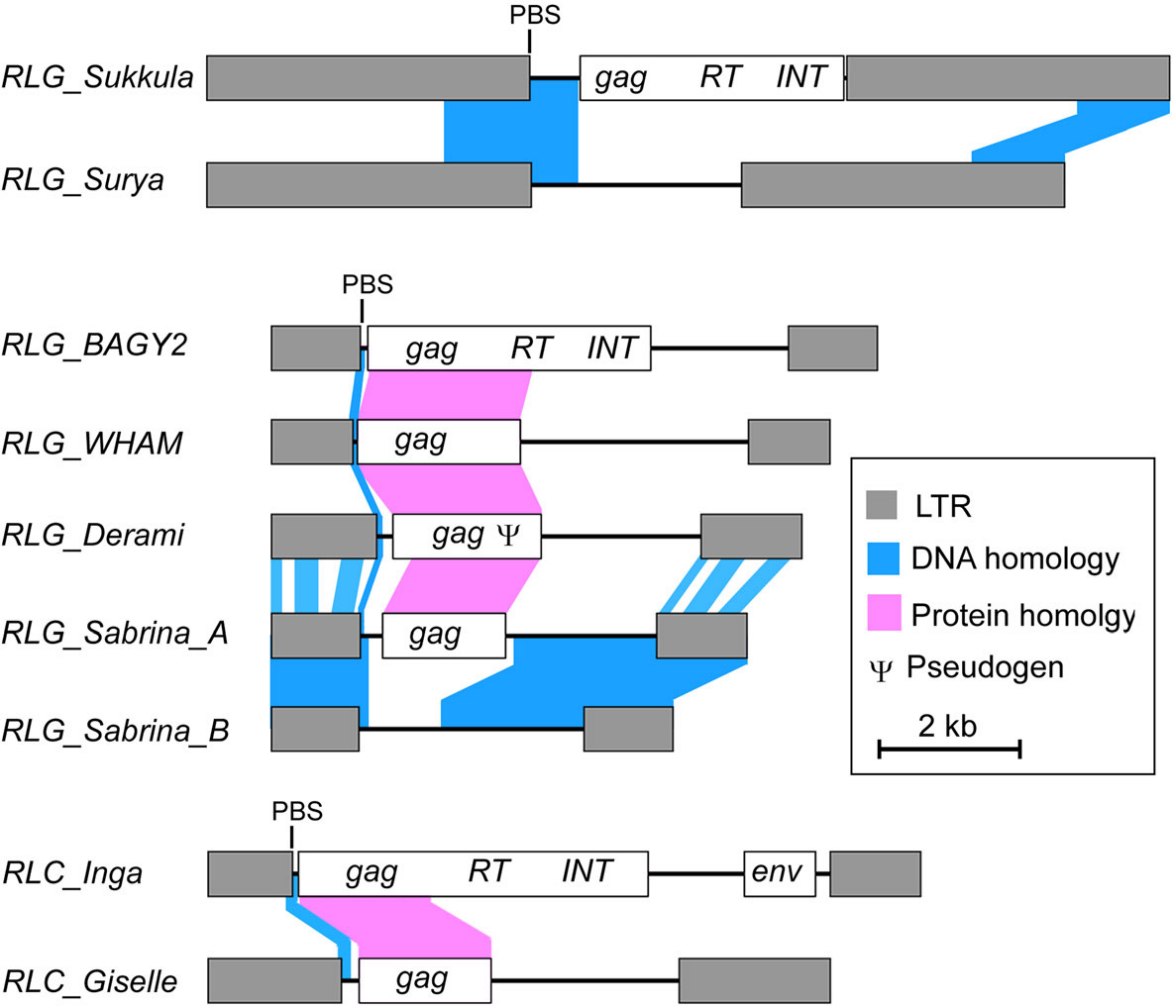
Sequence organization of non-autonomous LTR retrotransposon families and their putative autonomous partners. Sequence conservation between putative autonomous and non-autonomous partners is indicated with shaded areas. Regions of DNA homology are shown in blue, regions where predicted proteins show homology are shown in pink. PBS: Primer Binding Site

Interestingly, three of the top five TE families seem to be non-autonomous (*RLG_Sabrina*, *RLG_WHAM*, and *RLG_Surya*) because they have none or only fragments of the genes that are typically found in autonomous elements (Fig. 2). The second most abundant family even diverged into 2 subfamilies termed A and B (Fig. 2). For *RLG_Surya*, we suspect it is cross-mobilized by the much less abundant *RLG_Sukkula* family because of a strong sequence homology in their LTRs, which contain regulatory regions, and the region immediately downstream of the 5′ LTR, which contains the binding site for the tRNA primer (PBS) that initiates reverse transcription. Furthermore, *RLG_Sukkula* and *RLG_Surya* have similar chromosomal distributions, which one would expect if *RLG_Surya* elements are integrated into the genome by integrase proteins encoded by *RLG_Sukkula.* Such cross-mobilization has been described previously for barley *BARE2* elements [6].

**Fig. 3:**
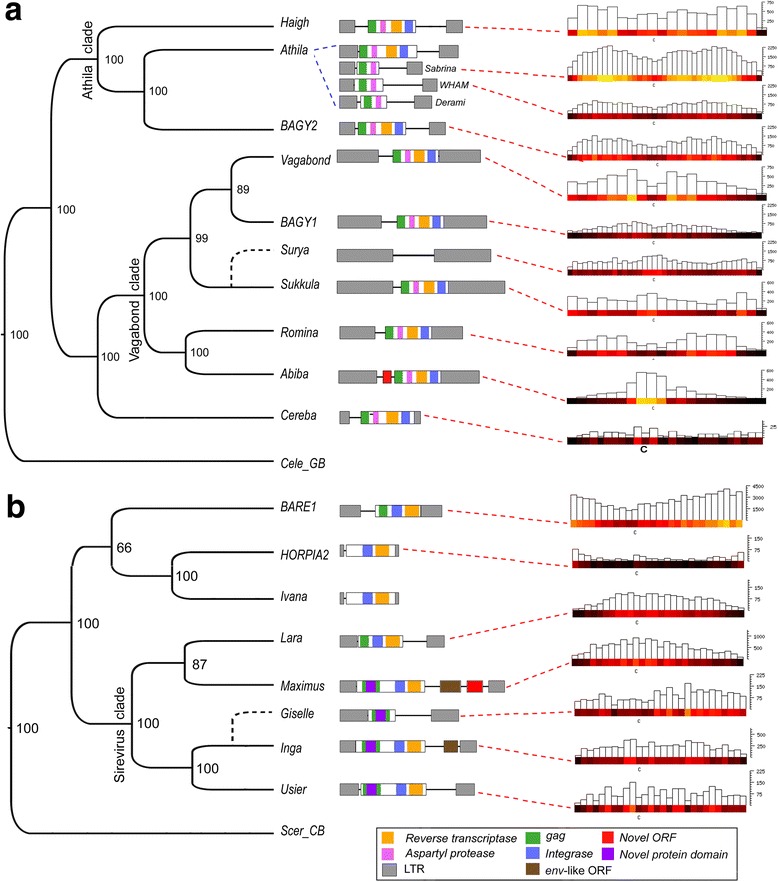
Distribution of *Gypsy* and *Copia* retrotransposons along barley chromosomes. Phylogenetic trees of the most abundant *Gypsy* and *Copia* families are shown at the left. For the construction of phylogenetic trees, the reverse transcriptase and integrase region of the predicted polyprotein was used. GAG (a structural protein forming the virus-like particle) and protease domains were excluded because they don’t show enough sequence conservation between families. The numbers at the forks indicate the number of times the group consisting of the families to the right of that fork occurred among the trees, out of 100 trees. Sequence organization and gene content of the individual retrotransposon families are shown in the center. Chromosomal distributions are shown at the right in bins of 20 to 40 Mb (depending on the copy number) as heat maps and bar plots to indicate absolute numbers. The y-axis indicates the total number of kb that is occupied by the respective TE family in each bin (Note that scales differ between families). Retrotransposon families with different evolutionary histories show different chromosomal distribution patterns. **a** Distribution of *Gypsy* elements on chromosome 2. **b** Distribution of *Copia* elements on chromosome 1

For *RLG_Sabrina* and *RLG_WHAM* (and the less abundant *RLG_Derami*), we could not identify any putative autonomous elements, but sequence similarity of their predicted, partial proteins suggests that their autonomous master elements are homologs of the *Athila* retrotransposon from Arabidopsis (*Athila* clade, Fig. 3). Possibly they are cross-mobilized by *RLG_BAGY2* which is the closest barley homolog of *Athila.* While sequence homology at the DNA level between *BAGY2* and the non-autonomous *RLG_Sabrina*, *RLG_WHAM* and *RLG_Derami* is limted to the 3′ termini of the LTR and the primer binding site (PBS), predicted GAG proteins show strong homology (Fig. 2). Overall structure and sequence homology between *RLG_Sabrina*, *RLG_WHAM* and *RLG_Derami* suggests that they all are descendants of a non-autonomous derivative of a *RLG_BARGY2*-like ancestor.

Also, the *Copia* family *RLC_Giselle* likely depends on closely related autonomous *RLC_Inga* family elements for transposition since *RLC_Giselle* does not have reverse transcriptase and integrase genes (Fig. 2). Interestingly, in all cases sequence conservation between autonomous elements and their proposed non-autonomous partners is highest in the region of the PBS. We therefore speculate that use of the same tDNA primer (to initiate reverse transcription) is a crucial factor for the functionality of non-autonomous elements. Additionally, the identified non-autonomous elements show different levels of degeneration: *RLG_WHAM*, *RLG_Sabrina_A* and *RLC_Giselle* all contain potentially intact *gag* genes and could therefore contribute at least in part to their own replication, while *RLG_Derami* has still a region homologous to *gag* but its ORF is interrupted by stop codons. Finally, *RLG_Suria* and *RLG_Sabrina_B* have completely lost all coding capacity (Fig. 2). These data indicate indicate that non-autonomous retrotransposons mobilized by a relatively small number of autonomous elements contribute substantially to barley genome size.

Non-autonomous derivatives are particularly numerous among Class II elements. Most dominant is the *Mariner* superfamily which is represented by at least 36 families. The top 10 *Mariner* families are all small non-autonomous elements ranging in size from 81 bp (*DTT_Athos*) to 274 bp (*DTT_Stolos* and *DTT_Pluto*, Table 1)*.* Such small *Mariner* elements are also referred to as *Stowaway* MITEs [16]. The most abundant *Mariner* family, *DTT_Thalos*, is present in more than 17,000 copies. Interestingly, we identified only about 150 potentially functional, autonomous *Mariner* elements. Thus, a vast number of non-autonomous DNA transposons is apparently relying on a very small number of functional master elements for their potential mobilization. The situation is similar for Harbinger transposons, but these elements are about four time less abundant (Table 1).

### Individual TE lineages occupy distinct chromosomal “niches”

*Gypsy* and *Copia* LTR retrotransposons are distributed throughout the chromosomes, as reflected in an even coverage of reverse transcriptase and integrase domains identified by PFAM (Additional file 1: Figure S2). However, at the individual family level, distributions vary strongly (Fig. 3). For example, the *Copia* element *RLC_BARE1* is enriched in distal regions of chromosome arms, as is the closely related but far less abundant *RLC_HORPIA2* (Fig. 3b). In contrast, *RLC_Lara* and *RLC_Maximus* are preferably found in proximal (peri-centromeric) chromosomal regions (Fig. 3b). Retrotransposon families of the *Gyspy* superfamily occupy complementary genomic niches: the interstitial regions of chromosome arms are dominated by families from the *Athila* clade (*RLG_Sabrina, RLG_WHAM* and *RLG_Derami*, Fig. 3a), whereas *RLG_Surya* and *RLG_Sukkula* are enriched in the proximal and distal regions. Generally, closely related families tend to have similar distribution patterns. An interesting exception is the *RLG*_*Abiba* family which is highly enriched in peri-centromeric regions, while its closest relative *RLG*_*Romina* shows a virtually inversed chromosomal distribution.

Among Class II elements, the proximal regions are occupied by the high-copy *CACTA* family *DTC_Balduin*, while families of the *Caspar* clade are strongly enriched in distal regions. Over 75% of *DTC_Caspar* elements are located in the terminal 20% of chromosome arms (see below), the strongest niche enrichment we found for any TE group (Additional file 1: Figure S3). For less abundant Class II superfamilies, such as *Mutator*, *Mariner*, or *Harbinger*, we observed a familiar pattern of enrichment in distal regions [9, 21, 22] (example in Additional file 1: Figure S4). However, here we have only considered long and putatively autonomous elements which contain at least large parts of a transposase gene. The vast numbers of short non-autonomous elements (MITEs) tend to cluster near genes [9, 15, 16, 21, 22] (see below), making their overall distribution largely congruent with that of genes. In general, individual TE families show nearly the same distribution patterns across all chromosomes (examples in Additional file 1: Figs. S5-S8), with only few exceptions where distribution patterns differ between chromosomes (see below).

### The space surrounding genes is a distinct genomic compartment

**Fig. 4:**
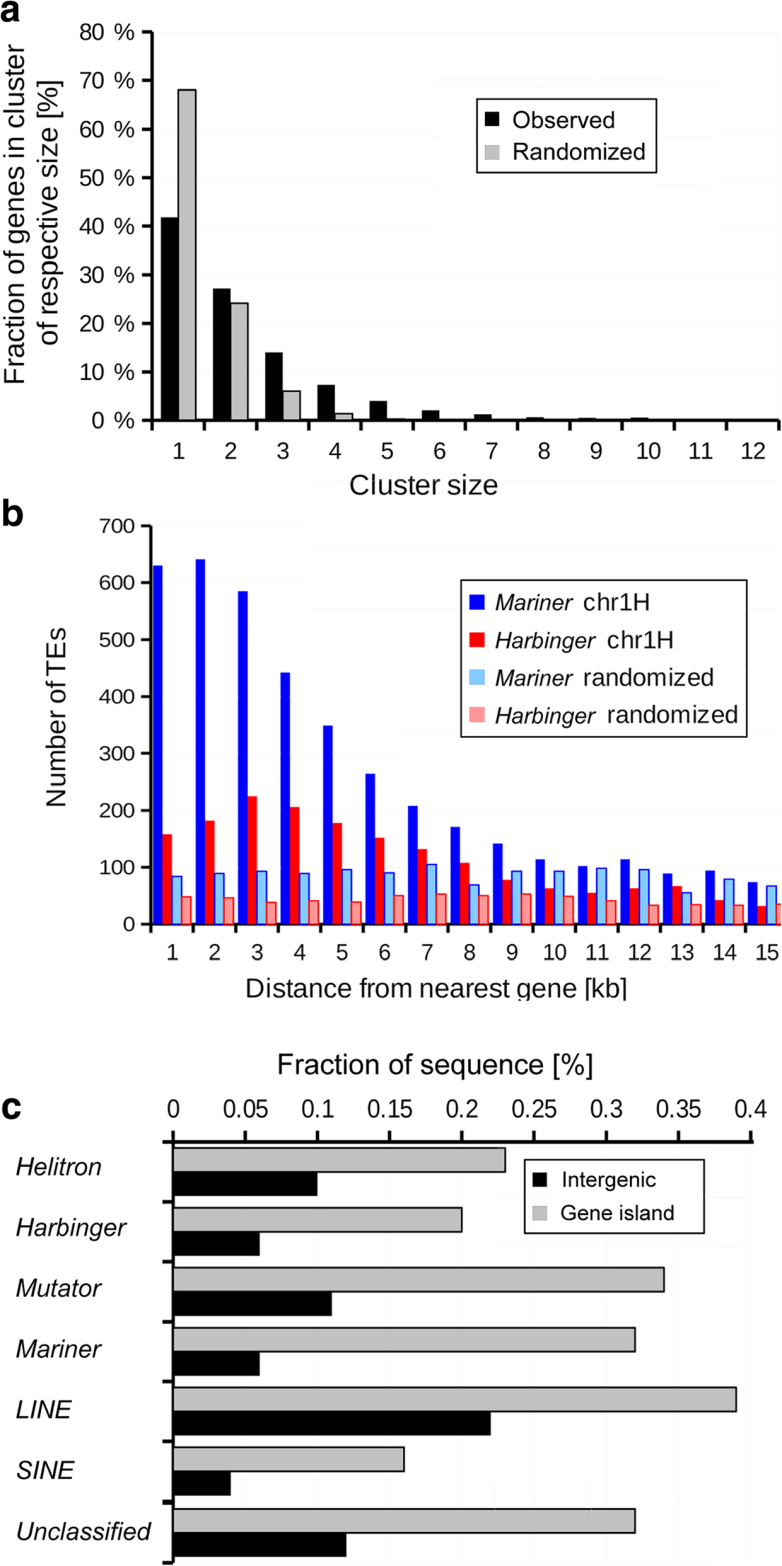
Characteristics of gene space **a** Fractions of genes found in clusters of different sizes. Almost 60% of the barley genes are found in clusters of 2 or more genes, in contrast to results of a simulation where genes are randomly distributed across the genome. In that case, only about 30% of the genes are found in clusters. **b** Distances of Mariner and Harbinger transposons to the nearest gene in the barley genome. The datasets for chromosome 1 show that Mariner elements prefer most to insert 1-2 kb away from genes. Here, we measured the distance from the middle of the annotated TE to middle of the nearest annotated gene. Note that for this analysis, we used only high-confidence genes of the HC1 level [4]. Thus the actual number of TEs near genes is likely to be higher. **c** Comparison of TE composition of gene islands with that of large (> 200 kb) intergenic regions

In addition to large-scale TE niches, gene space represents a unique genomic compartment with its own TE “environment”, largely independent of the gene location along the chromosomes. Genes tend to be enriched in distal chromosomal regions in barley, with gene density forming an exponential gradient from centromeres to telomeres [4]. In addition to this gradient along chromosomes, genes are distributed non-randomly. They are found mostly in clusters of two to seven genes, (we defined genes that are separated by less than 20 kb as belonging to the same cluster). Individual clusters are separated by “seas” of repetitive DNA (Fig. 4, Additional file 1: Figure S9). Additionally, the TE landscape close to genes differs strongly from that of intergenic regions (here, we arbitrarily defined as “intergenic regions” stretches of at least 200 kb that do not contain genes, Fig. 4b and c). As mentioned previously, close to genes, we find mostly small, non-autonomous DNA transposons. More than a third (36%) of *Mariner* and 25.7% of *Harbinger* transposons are found within 5 kb of genes, a highly significant enrichment. Within 10 kb, this enrichment increases to almost 50% of *Mariner* and over 40% of *Harbinger* elements (Fig. 4b). As previously described [4], LTR retrotransposons are strongly under-represented in the 1-2 kb upstream and downstream of genes. In the following, we present separate analyses of how Class I (retrotransposons) and Class II (DNA) transposons contribute to the genomic environment of genes.

### The retrotransposon neighbors of genes

**Fig. 5:**
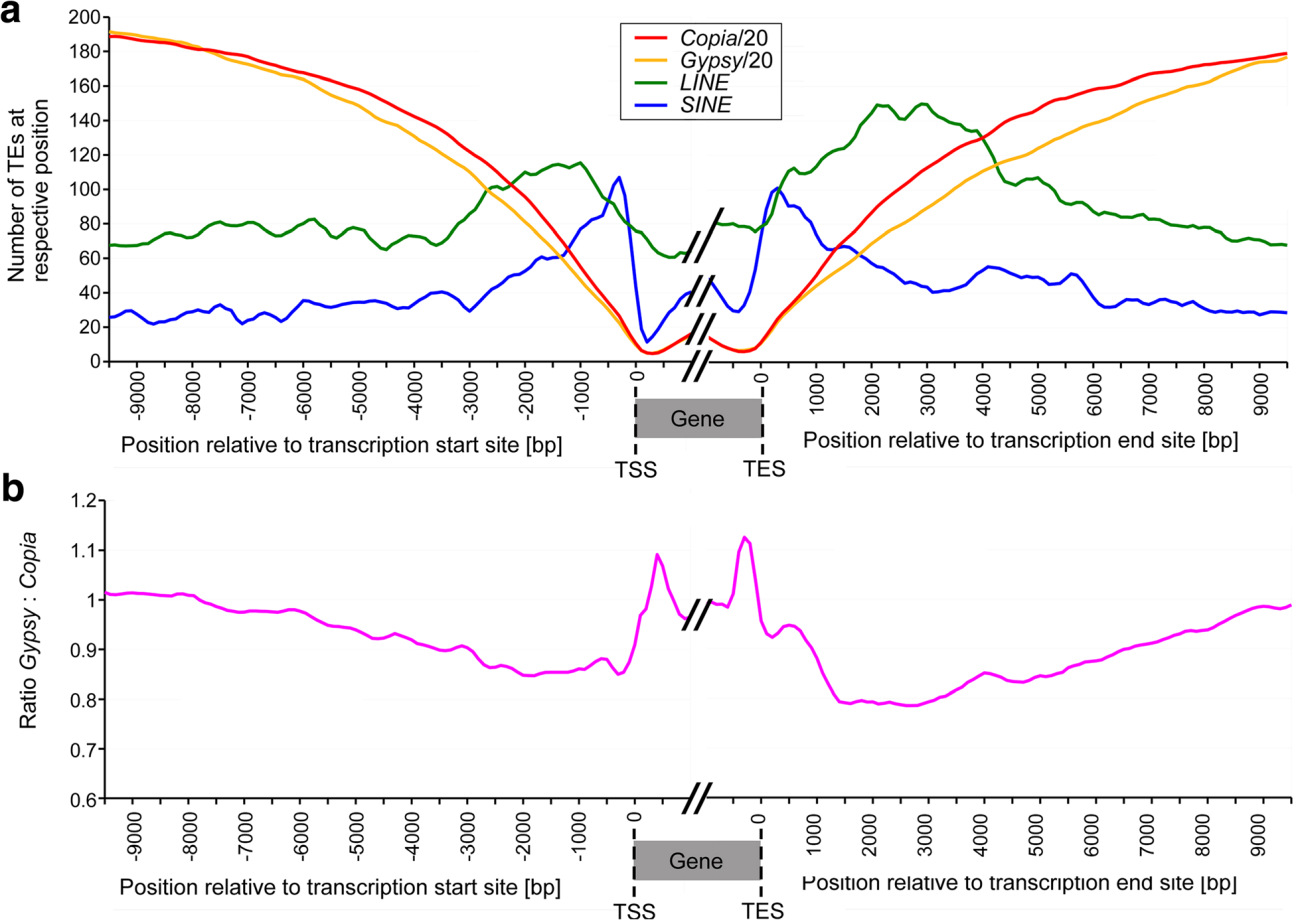
Frequencies of retrotransposons in the vicinity of genes. The plots are anchored around transcription start sites (TSS) and end sites (TES) of 28,316 high-confidence genes. **a** Overall frequencies of LTR retrotransposons (*Gypsy* and *Copia* elements), LINEs and SINEs. Note that values for *Gypsy* and *Copia* elements are divided by a factor of 20 to fit into the plot. **b** Ratio of *Gypsy* vs. *Copia* retrotransposons near genes

In addition to being enriched in specific niches on a chromosome-wide scale, retrotransposons also show distinct patterns of distribution in the vicinity of genes (Fig. 5). In the 10 kb upstream of the TSS of 28,316 high-confidence genes, we identified 179,137 retrotransposons, 97.6% (174,995) of which are LTR retrotransposons, while only 470 are SINEs (0.26%) and 3672 are LINEs (2.05%, Additional file 1: Table S1). The situation is similar downstream of genes, where we identified a total of 170,123 retrotransposons insertions within 10 kb of the transcription end site (TES). Here, SINEs and LINEs contribute slightly (but not significantly) more to the retrotransposon population (591 or 0.35% and 4108 or 2.4%, respectively).

Of particular interest are retrotransposon insertions that are very near genes. Here, we focused on retrotransposons that are inserted within 500 bp of the TSS and TES (Additional file 1: Table S2). Interestingly, retrotransposon composition changes drastically near genes: starting approximately 3 kb upstream of the TSS and 5 kb downstream of the TES, LINEs, and SINEs are found more frequently, while the frequency of LTR retrotransposons (i.e. *Gypsy* and *Copia* elements) drops sharply (Fig. 5a). SINEs are found approximately four times more frequently immediately up- and downstream of genes than at distance of 10 kb (Fig. 5a). Also LINEs are more frequent near genes. Additionally, LINEs show an asymmetric distribution with a higher frequency downstream of genes (Fig. 5a). These data suggest that both SINEs and LINEs may have a preference to insert near genes.

The previous study based on the barley genome sequence reported a genome-wide average ratio of 1.3 of *Gypsy* vs. *Copia* retrotransposons [4]. Toward genes, the *Gypsy*: *Copia* ratio steadily decreases (Fig. 5b). At a distance of 10 kb from genes, the *Gypsy*: *Copia* ratio is approximately 1.1, close to the genome-wide average of 1.3. This ratio reaches a minimum of 0.82 at approximately 800 bp upstream of the TSS. Similarly, the *Gypsy*: *Copia* ratio has a minimum of 0.77 approximately 2000 bp downstream of genes. Curiously, the *Gypsy*: *Copia* ratio spikes sharply after the TSS and TES inside genes to near the genome-wide average (Fig. 5b), suggesting that *Gypsy* elements are deleterious in up- and downstream regions of genes.

Of the TEs that are inserted within 500 bp upstream of genes, LINE elements were significantly enriched in forward orientation relative to their nearby genes (Additional file 1: Table S2), while they were enriched for reverse orientation downstream of genes, except within 100 bp of the gene, where the trend reversed (as shown by scanning downstream regions in a sliding window of 100 bp, Additional file 1: Figure S10). These data suggest that there is selection for transcriptional orientation of some retrotransposon superfamilies relative to genes. However, the signals are relatively weak and we remain cautious as to the conclusion that can be drawn from these data.

### Barley gene space is characterized by distinct DNA methylation patterns.

**Fig. 6:**
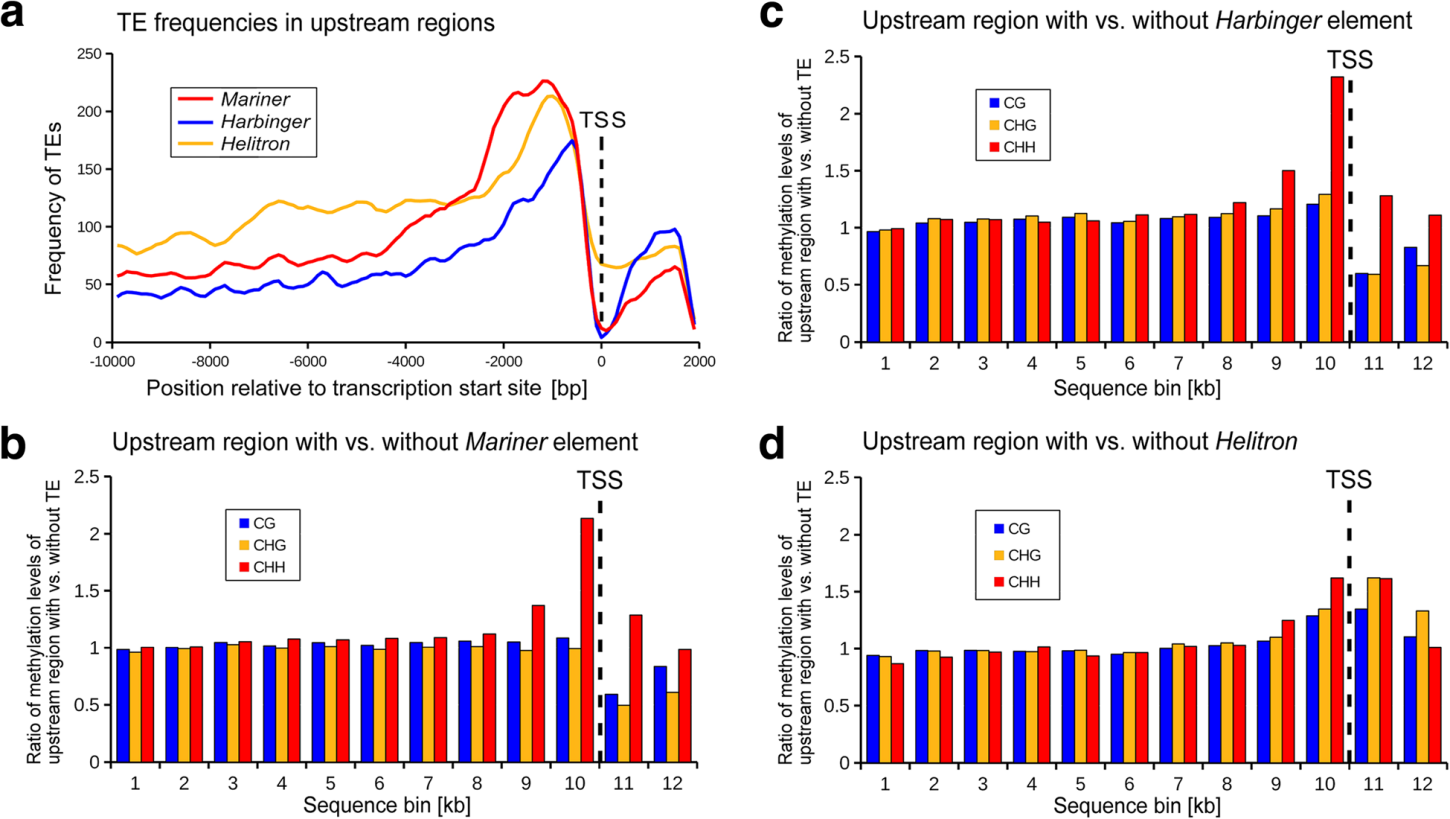
Methylation levels in upstream regions of genes. **a** Frequency of *Mariner*, *Harbinger* and *Helitron* elements in the region 10 kb upstream and 2 kb downstream of the predicted transcription start site (TSS). **b** Relative methylation levels surrounding the TSS calculated in bins of 1000 bp. We compared average methylation levels of 1763 genes that contain a *Mariner* element in the upstream region with those of genes that do not have such elements in their upstream region. **c** Same as in (**b**) with 759 genes with *Harbinger* elements. **d** Same as in (**b**) with 506 genes with *Helitrons*. Relative methylation levels in CG and CHG sites levels drop sharply while CHH levels are higher in the bins surrounding the TSS for genes that contain *Mariner* and *Harbinger* elements. This effect cannot be observed for *Helitrons*

Small non-autonomous DNA transposons of the *Mariner*, *Harbinger* and *Helitron* superfamilies are preferably inserted immediately upstream of the predicted transcription start site (TSS, Fig. 6a). As TEs are known to be targets of epigenetic silencing [23], especially in grasses [24], we focused on genes with TEs between 1500 bp upstream of the TSS to 500 bp downstream of it (we reasoned that TE insertions in this region are likely to affect regulatory elements of genes). We hereafter refer to this region as the “promoter”. We explored how TE insertions could potentially affect nearby genes by analyzing local methylation levels revealed by bisulphite sequencing of seedling leaf DNA. Analyzing high-confidence genes from chromosome 1H, 2H, and 3H, we identified 1763 genes that contained *Mariner* elements, 759 genes with *Harbinger* elements, and 506 genes with *Helitrons* in this region. There are an additional 14,114 genes that do not contain any of these elements in the promoter region (the analysis was restricted to chromosomes 1H, 2H, and 3H due to computational limitations)*.* We examined methylation levels per kb for the region encompassing 10 kb upstream of the TSS and ending 2 kb downstream of the TSS inside the genes (Additional file 1: Figure S11).

In general, methylation levels in CG and CHG sites decrease sharply near genes, reaching a minimum at the TSS. Downstream of the TSS, CHG methylation levels increase only slightly, despite a sharp increase in GC content (and thus potential methylation sites) which is typical for genes in grasses [25]. CG methylation levels increase much more strongly again after the TSS (Additional file 1: Figure S11). In contrast, CHH sites, which are more frequent in the genome than CG and CHG sites, are generally less methylated and show only a slight increase upstream of the TSS (Additional file 1: Figure S11). This in agreement with previous findings in maize [26]. However, methylation levels differ between genes with and without DNA transposons in their promoters (see below).

### Class II transposon insertions are associated with altered methylation levels

To study whether the presence of specific TE types is associated with differences in methylation levels, we separately analyzed genes which contain no TEs and those with *Mariner*, *Harbinger*, or *Helitron* elements in the region 10 kb upstream to 2 kb downstream of the TSS of genes (Additional file 1: Figure S11). We divided the 12 kb regions into bins of 1000 bp and calculated average methylation levels for each bin. We then calculated for each bin the ratio of methylation levels of genes without transposons in their promoters with methylation levels of genes with transposons in their promoters. Interestingly, methylation levels of CG and CHG sites in promoters that contain *Mariner* or *Harbinger* transposons are on average approximately 50% lower than those of promoters without such elements. This effect can be seen in the sequence bins nearest the TSS, while further upstream of the genes, methylation levels are very similar between genes with and without transposons in their promoters (Fig. 6b and c). Two possible explanations for these findings are: first, insertions of *Mariner* or *Harbinger* transposons suppress subsequent DNA methylation; alternatively, *Mariner* and *Harbinger* elements simply target open chromatin (i.e. genes with high expression levels, usually correlated with low methylation levels).

The situation is different for CHH sites, where methylation levels are higher, especially for the 1 kb regions immediately upstream of the TSS, if *Mariner* or *Harbinger* elements are present in the promoter (Fig. 6b and c, Additional file 1: Figure S11b and S11c). This is consistent with previous findings in maize, where the presence of small DNA transposons was also found to be associated with elevated methylation levels in CHH sites [26]. However, association of *Mariner* or *Harbinger* elements with decreased CG and CHG levels have, to our knowledge, not been reported. RNA-directed methylation silences transposable elements in plants [27]. Thus, on one hand, promoters containing *Mariner* and *Harbinger* elements tend to have lower CG and CHG methylation levels and, on the other hand, they are associated with higher levels of CHH methylation. At this point, we do not have enough data to determine the effect of this dichotomy on gene functions. We are therefore also hesitant to make cause-and-effect conclusions. It is possible that, in some cases, changes in methylation occurred independently before or after the TE insertions.

In contrast to *Mariner* and *Harbinger* elements, methylation levels of genes that contain *Helitrons* in their promoters differ only very little from those genes without such elements (Fig. 6d, Additional file 1: Figure S11d). Moreover, CG, CHG and CHH methylation levels all show a very similar pattern of a slight (approximately 50%) increase near the TSS. Since TEs are known to influence expression of nearby genes [28], we wanted to test whether the observed differences in methylation levels can be associated with expression levels of genes. Thus, we studied barley gene expression data from embryonic, leaf and root tissue. Additionally, we examined expression data from roots in 17 and 28-day-old plants. We found that the number of genes that show no transcription at all in the four transcriptome datasets is significantly higher in genes that contain *Helitrons* in their promoters than in genes without TEs in their promoters (Additional file 1: Figure S12). Other than that, we found no significant differences in expression levels of genes with or without *Mariner*, *Harbinger*, or *Helitron* transposons in their upstream region (Additional file 1: Figures S13 and S14).

### Target site preference of TEs

**Fig. 7:**
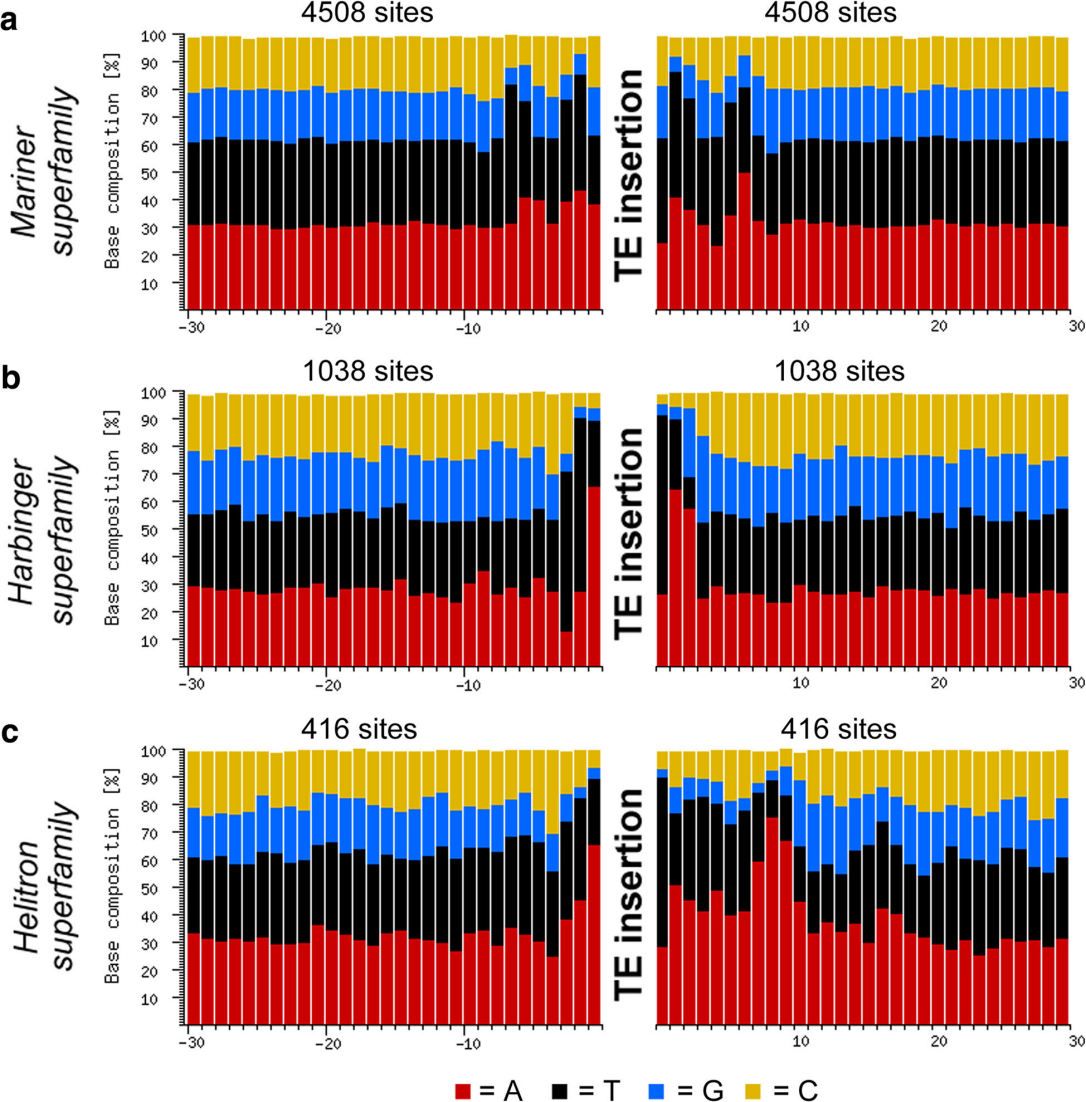
Target site preferences of high-copy Class 2 transposons from barley. For the plots, the 30 bp flanking complete elements (i.e., not truncated) on both sides were collected. Then the different nucleotides at each position were counted across across all insertion sites of a given TE type. The x-axis is the bp position relative to the TE insertion site, while the y-axis shows the relative nucleotide composition for each position. **a** *Mariner* elements have a strong preference for A/T dinucleotides 2 and 5 bp away from the insertion site, while (**b**) *Harbinger* elements almost invariable prefer 3 bp A/T -rich motifs. **c** Interestingly, *Helitrons* have a preference for an asymmetric target, strongly preferring an AAA motif 8 bp downstream of the insertion site

We analyzed the insertion sites of several high-copy TEs, including *RLC_BARE1* and *RLG_Sabrina*, as well as multiple families of *Mariner, Harbinger*, and *Helitron* elements. Here, we only used TE copies where both ends were intact to assure that we indeed only analyze the sequences precisely flanking the individual insertions. Interestingly, we observed pronounced differences in target site preference (Fig. 7). Class II elements target very specific motifs: *Mariner* elements prefer A/T-rich targets with the consensus [T/A][T/A]nnT-Ann[T/A][T/A], where the dash represents the insertion site (Fig. 7a), whereas *Harbinger* transposons prefer a short TAA motif (Fig. 7b). Interestingly, *Helitrons* have a preference for an asymmetric target, as their insertion sites are highly associated with an AAA triplet starting 8 bp downstream of an A-T insertion site (Fig. 7c). In contrast, we could not detect clear target site preferences for Class I elements: the high-copy LTR retrotransposon *RLC_BARE1* has only a weak preference for G/C 7-8 bp away from the insertion site, while *RLG_Sabrina* has a slight preference for GGG motif 3-4 bp upstream of the insertion site and a CC motif 4 bp downstream (Additional file 1: Figure S15).

**Fig. 8:**
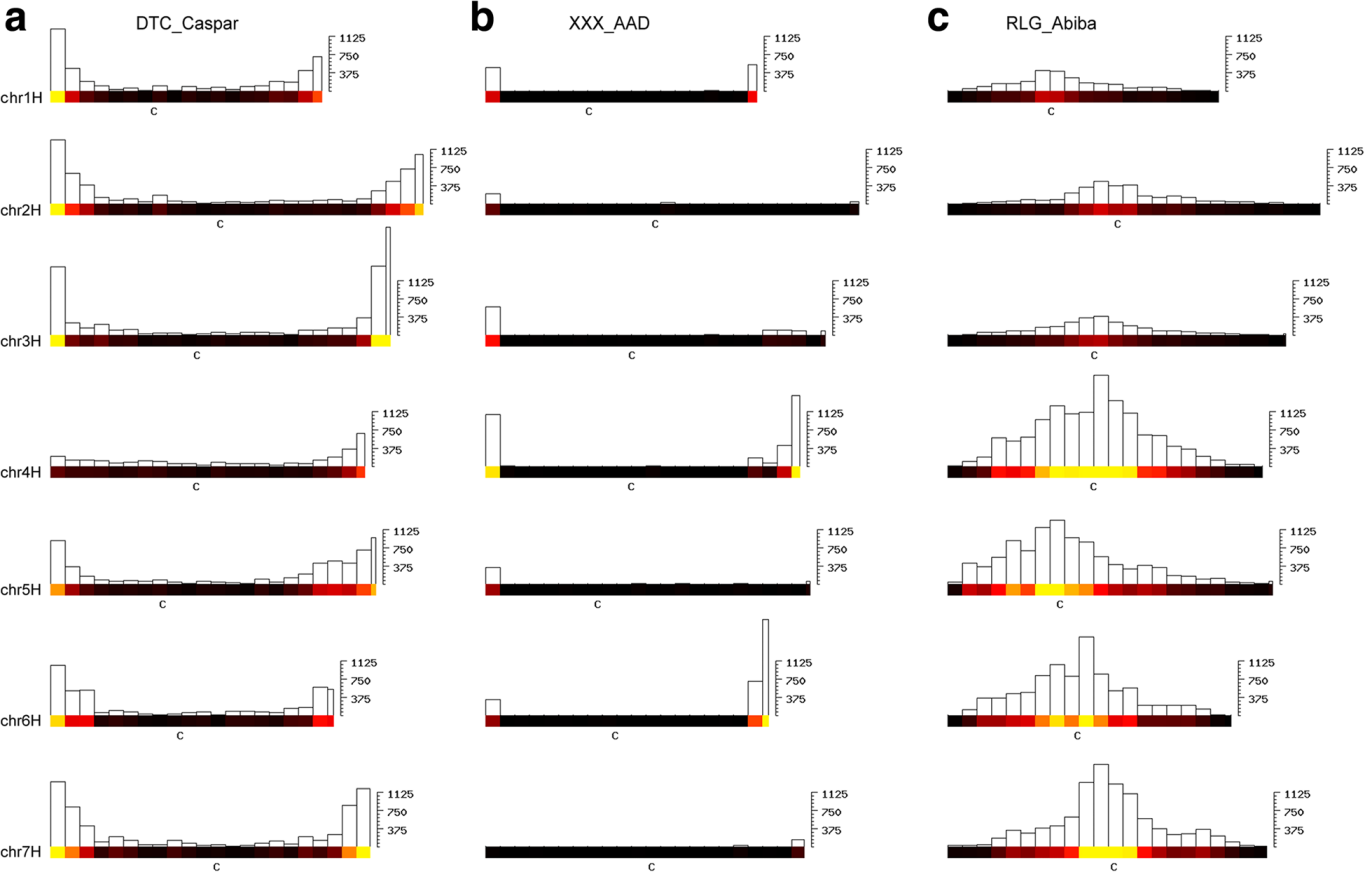
Examples for TE families with varying distributions between chromosomes. Chromosomal distributions are shown at the right in bins of 30 Mb (depending on the copy number) as heat maps and bar plots to indicate absolute numbers. The y-axis indicates the total number of kb that is occupied by the TE family in each bin (Note that scales differ between families). **a** The *CACTA* family *DTC_Caspar* is depleted on chromosome 4H, especially on its short arm. **b** The tandem repeat family *XXX_AAD* is highly enriched in the majority of telomeric regions, but practically absent from telomeres of 2HL, 5HL and 7HS. **c** The *Gypsy* family *RLG_Abiba* is generally enriched in cetromeric and pericentromeric regions, but its overall abundance differs strongly between chromosomes

Interestingly, some TE families also show varying distribution patterns between chromosomes (Fig. 8). For example, the *CACTA* family *DTC_Caspar* is generally highly enriched in distal chromosomal regions. However, it is nearly absent from the telomeric region of the short arm of chromosome 4H (Fig. 8a). Also the tandem repeat family *XXX_AAD* (for which we do not know how it is replicated) is highly enriched in telomeric regions of several chromosomes, but virtually absent from others (Fig. 8b). Finally, the *RLG_Abiba* family shows strong difference in abundance between different chromosomes as it is 4-5 times more abundant on chromosomes 4H through 7H than on chromosomes 1H through 3H (Fig. 8c). At this point we have no explanation as to what might cause this differential distributions.

## Discussion

The repetitive landscape of the barley genome is completely dominated by a handful of LTR retrotransposons with extremely high copy numbers. Despite the large difference in size between the barley genome and smaller plant genomes, TE diversity is similar: The relatively small *Brachypodium* genome (275 Mbp) went through a very detailed repeat annotation, leading to the identification of over 170 different TE families [9]. By comparison, we identified less than twice as many TE families in barley, although the barley genomes is almost 20 times larger than the *Brachypodium* genome. Thus, the factor that determines genome size is the copy numbers of the most abundant families.

The roughly 350 TE families identified by us cover 80.8% of the assembled sequence [4]. Considering that gene space contributes only 2-3% to the genome, approximately ~16% remained un-annotated. This proportion of un-annotated sequence is comparable to other genomes. In maize, approximately 12% remained un-annotated [7], while in *Brachypodium*, un-annotated sequences make approximately 25% of the genome [9]. We assume that these un-annotated portions of these genomes contain additional, yet uncharacterized, TE families. These could be highly degenerated TEs, or exotic TE types that have very low copy numbers and thus escape detection. Indeed, in-depth analysis of retrotransposon diversity in maize showed that many families of the > 400 retrotransposon familes are present in only a handful copies [10]. Thus, if the complexity of the repetitive fraction of the barley genome is similar to that of the maize genome, one has to expect that hundreds of low-copy TE families still remain to be discovered in barley.

Interestingly, small non-autonomous TEs such as MITEs are present in similar copy numbers as in smaller genomes. Both rice and Brachypodium contain roughly 25,000 MITEs, while we identified approximately 54,000 such elements. We assume that this has to do with the fact that MITEs are enriched near genes [15–19] and gene numbers are very similar in all plant genomes.

### TEs divide the barley genome into distinct compartments

The key finding of our current analysis was that the barley genome is highly compartmentalized with respect to the localization of different types of TEs. It is a well-described phenomenon that differences in TE insertion mechanisms can lead to compartmentalization of genomes by TEs [29]. Previous studies also indicated that the distribution of TEs is also the result of retention bias, i.e. selective pressure that restricts their accumulation in genomic regions where they are best “tolerated” [30]. Combination of these factors can lead to very distinct distribution patterns, especially in repeat-rich large plant genomes such as the one of maize [10, 11]. However, the level of diversity in distribution patterns of different TE families in barley still came as a surprise to us. Indeed, we find that the genomic localization of TEs is strongly associated with both their phylogeny and their target site preference (see below). This suggests that the distribution of individual TE families is to a large degree the result of their genetic composition which allows them to target preferred genomic compartments.

However, one could still argue that TE insertions are in principle random but that family-specifc distribution patterns emerge because they are removed from the genome at different rates in different chromosomal regions. For LTR-retrotransposons, we consider this hypothesis unlikely because these elements all have similar sizes and sequence compositions. Nevertheless, retention bias could play a role in the case of *CACTA* elements because these elements usually contain large regions of low-complexity DNA, tandem repeat arrays and widely ranging G/C contents [13]. Such sequences can be hot spots of double-strand breaks and subsequent rearrangements or deletions (reviewed by [31]). Additionally, it is likely that retention bias plays a role for TEs that inserted near genes. For example, LINEs could be more abundant downstream of genes than upstream simply because insertions in gene promoters are more likely to be deleterious than insertions in the downstream region.

### Insertion preference could be driven by target sequence motifs

Niche specificity could arise from sequence-dependent target site preferences of the respective transposase or integrase enzymes. Indeed, we found that especially small non-autonomous elements of the *Mariner* and *Harbinger* superfamilies have a strong preference for A/T-rich targets. The fact that *Mariner* elements almost invariably prefer a TA target site while harbinger elements prefer TAA targets has been described before [15, 16]. However, our data indicate that the motif which is actually recognized by the *Mariner* transposase is an A/T rich 10 bp motif with the TA target at its center (Fig. 7a). Such motifs (e.g. TATA boxes) occur frequently in promoters. This target preference could, in part, explain their preference for promoter sequences. Alternatively, these elements might simply target open chromatin (i.e., transcriptionally active) regions during transposition and establish themselves close to genes because their small size does not disrupt promoter function.

Particularly interesting is the preference of *Helitrons* for an asymmetric target with an AAA triplet starting 8 bp downstream of an A-T insertion site. Previous studies reported the preference of *Helitrons* for a 5′-AT-3′ insertion site [5] and for generally A/T rich sequences [32]. However, preference for an asymmetric target has, to our knowledge, not been reported for any type of TE. The asymmetric sequence composition of the target site suggests that the helicase/recombinase protein of *Helitrons* binds the target DNA at the insertion site as well as one rotational period away in the DNA double-helix (i.e. 10 bp).

### Niche specificity my be encoded by the TEs themselves

In contrast to DNA transposons, we found no distinct sequence-based target site preference for LTR retrotransposons. However, our analysis was limited to two high-copy families where we could extract a sufficiently high number of full-length copies. Indeed, previous studies have reported a preference for short palindromic sequences in Sireviruses [11]. This specific clade of *Copia* elements is represented in barley by less abundant TE families (Fig. 3b) for which we could not identify enough full-length copies to perform a quantitative analysis of target sites.

Despite the lack of obvious target sequence specificities, different LTR retrotransposon families show very distinct chromosomal distributions. This suggests that their integrase enzymes target epigenetic patterns, such as histone modifications, rather than DNA sequence motifs. Previous studies reported that *RLG_Cereba* retrotransposons are particularly enriched in peri-centromeric regionss [33], as are its homologs (the CRM elements) in maize, rice, and *Brachypodium* [7, 9]. However, for barley we could not confirm such enrichment (Fig. 3a). Instead, we found that the *Abiba* family has taken over the proximal (peri-centromeric)” niche” in barley. We speculate that its unique preference for centromeric regions may be due to the product encoded by an ORF that is not found in any other retrotransposon family (Fig. 3a). This protein might have novel properties that enable *Abiba* elements to specifically target centromeric regions, potentially similar to previously described targeting domains of integrases. For example, chromodomains in integrase proteins of CRM elements that likely target centromere-specific histone modifications [34]. Retrotransposons have been shown to have a wide range of targeting mechanisms. For example, the yeast Ty1 integrase interacts with the AC40 subunit of RNA polymerase III (Pol III) which leads to insertions upstream of Pol III-transcribed genes [35]. Similarly, Ty5 *Copia* retrotransposons from yeast encodes an integrase with a domain that targets the silent information regulator 4 Sir4p, a heterochromatic protein at chromosome ends [36, 37]. An interesting variation are the telomere-specific LINE retrotransposons *TAHRE*, *TART*, and *HeT*-*A* in *Drosophila melanogaster*. These retrotransposons apparently target the 3′ OH of the DNA at chromosome ends [37] and have taken over telomerase function in *Drosophila*. Considering these previous findings, we speculate that the observed niche specificity of many of the barley TE families is driven by affinity of integrase proteins to specific histones or their modifications. This might also be the case for transposase proteins of *CACTA* elements where different families also show different niche preferences. However, further studies involving wet lab experiments will be necessary to precisely identify the molecular mechanisms of how TEs target their preferred genomic niches in barley.

## Conclusions

Barley provided unique insights into the structure and organization of a plant genome near to the average size of those of the angiosperms. Previous analyses of TE content and composition in such genomes have been limited to general abundances, largely due to the absent, or poorly assembled, intergenic sequences. The near complete chromosome assemblies of barley allowed for a detailed analysis of abundance and chromosomal distribution of individual TE families. Our findings emphasize the importance of TEs as active contributors to the evolution of genomes.

## Methods

### TE annotation and copy number estimates

Basis for all analyses was the TE annotation produced in the framework of the international barley sequencing consortium (IBSC) [4]. For this study, we used an additional approach to precisely identify the boundaries of full-length elements (i.e. ends that are not truncated) for the characterization of populations of high-copy TE families. This annotation approach was complementary to that used by Mascher et al. [4] (which should still be used as the reference TE annotation). In our approach, chromosomes were split into short segments of 180 bp, which were used in blastn searches against the TREP database (www.botinst.uzh.ch/en/research/genetics/thomasWicker/trep-db.html). This was done to allow precise annotation of the short segments, especially the identification of TE boundaries. In a second step, the annotations of the individual segments were combined. Since TEs often contain divergent regions that do not align well with the reference TE, gaps of less than 100 bp between blastn alignments were bridged, if the same TE family in the same orientation was found on both sides of the gaps. Additionally, TEs often contain problematic motifs that cause gaps in the sequence. Thus, if a gap was found within 80 bp of an annotated TE, the stretch between TE and gap was annotated as belonging to the same TE.

For TE classification and nomenclature, we applied the classification system by Wicker et al. [5]. Here, TE family names are preceded by a three-letter code that represents the TE superfamily (e.g., RIX for LINEs, RSX for SINEs, RLX for LTR retrotransposons, RLG for *Gypsy* LTR retrotransposons, and RLC for *Copia* LTR retrotransposons). Genome size data for angiosperm plants were obtained from the Angiosperm DNA C-values database (data.kew.org/cvalues).

### Phylogenetic analysis

In this study, we used the definition of family proposed by Wicker et al. [5].TEs belong to the same family if their DNA sequences are over 80% identical and can be aligned over > 80% of their length. However, we complemented this definition with phylogenetic analyses. Phylogenetic analysis of *Gypsy*, *Copia* and *CACTA* elements was performed on predicted protein sequences deposited at the TREP database (botinst.uzh.ch/en/research/genetics/thomasWicker/trep-db). Protein domains in predicted ORFs were identified with PFAM (pfam.xfam.org), SignalP (cbs.dtu.dk/services/SignalP), and COILS (embnet.vital-it.ch/software/COILS_form.html). For the construction of phylogenetic trees of *Copia* and *Gypsy* elements, the reverse transcriptase and integrase region was used, while for *CACTA* elements, the predicted transposase protein was used. Protein sequences were aligned with Clustalw and the phylogenetic tree was constructed with MrBayes (mrbayes.sourceforge.net) using standard parameters with 10,000 generations.

For TE content analysis in up- and downstream regions of genes, the 10 kb immediately flanking the predicted coding sequences (CDS) of 28,316 HC1 high-confidence genes were extracted from the genome assembly (for definition on high-confidence genes, refer to Mascher et al. [4]). The genomic segments were then used in blastn searches against the TREP database. After an initial annotation, previously unclassified or poorly characterized TE families were re-analyzed and new consensus sequences were constructed. For construction of consensus sequences, we used up to 100 (as many as possible, but at least 3) full-length copies for individual TE families. These were aligned with Clustalw. The consensus sequence was then generated from the multiple alignment. If subfamilies were present, we constructed consensus sequences for individual subfamilies if a sufficient number of full-length copies could be identified.

Analysis of up- and downstream regions was then repeated with the updated TREP database. Based on blast outputs, it was determined, for every 20th base position of the 10 kb segments, which TE family produced the longest blastn hit at that respective position. This resulted in 500 data points for each up- and downstream region of the 28,316 genes. The resulting matrix was used as basis for the plots shown in Figs. 5a and 6a. This approach was used in a previous study [19] and was taken because it allows a rapid assessment of TE contents of up and downstream regions independent of existing TE annotation.

For TE vs. gene orientation (Additional file 1: Tables S1 and S2, Figure S11), the annotations, CDS orientations and start and end points, and TE annotation and start and stop points were taken from Mascher et al. [4]. TE orientation vs. number was then plotted for a sliding window of 100 bp moved in 1 bp increments. Significance of enrichment of TE in up- and downstream regions of, as well as bias in transcriptional orientation was tested with a Chi-Square test.

### Methylome library preparation and sequencing

DNA was isolated from barley seedling leaves using the CTAB method [38], and 2 μg DNA was used to prepare the sequencing library. Briefly, DNA was sheared to 200-300 bp fragments, followed by end repair, A-tailing, adapter ligation, and dual-SPRI size selection (250 bp – 450 bp) according to the manufacturer’s instructions (KAPA library preparation kit, KK8234). The library was then treated with bisulfite to convert unmethylated cytosine to uracil using Zymo EZ DNA methylation lightning kit (D5031). The converted DNA was then amplified using KAPA HiFiHotStart Uracil + (KK2801) with the following program: 95C for 2 min, 7 times of 98C/30s, 60C/30s, 72C/4 min, a final extension at 72C for 10 min. The PCR products were cleaned using Beckman SPRI beads.

The library size was checked using an Agilent Bioanalyzer to make sure that it was in the right range (200-700 bp, with a peak around 300 bp). It was quantified using qPCR to ensure that it met the sequencing criteria (> 2 nM). The library was then sequenced on two Illumina lanes using a HiSeq2500. A total of 478,688,629 paired-end 125 bp reads were generated.

### Methylome mapping in 100 bp non-overlapping sliding tiles

The adapter sequences were trimmed and read quality was assessed using Trim_glore under the paired-end reads mode. After quality control, 473,730,433 read pairs were kept. These reads were then mapped to the barley genome (Version 160,404) using BSMAP (version 2.90), allowing at most 5 mismatches. Because the barley genome is very big, we divided the genome into two files for mapping purposes. The first file contains chromosomes 1-4 and the other file contains chromosomes 5-7. Within each file, the chromosomes were also divided into two parts, because the entire chromosome size is too big to be aligned. For each alignment (chromosomes 1-4 and 5-7), we only kept reads that are properly paired and that are uniquely mapped. After alignments, the two output BAM files were merged and only the reads that were uniquely mapped for all seven chromosomes were kept. These left 234,762,441 read pairs. Those reads were then used to extract methylation information at individual cytosine sites using methratio.py (BSMAP). The output file from methratio.py was used to calculate methylation levels at 100 bp non-overlapping sliding windows across the barley genome for each of the three sequence contexts, CG, CHG and CHH (H = A, C or T) using custom scripts. The methylation levels were calculated using the formula #C/(#C + #T) for each context (CG, CHG, CHH) for all sites within each 100 bp window. Essentially, this determines the count of sites that are methylated and divides by the total count of covered sites in this region. This provides a proportion of methylated sites for each context for each 100 bp and we have not applied a coverage criteria. Barley transcriptome data was obtained from IPK Gatersleben, Germany (http://barlex.barleysequence.org).

## Additional file

Additional file 1: Supplementary Tables and Figures. (PDF 3776 kb)

## Abbreviations

CDS: Coding sequence
IBSC: International barley sequencing consortium
INT: Integrase
LINE: Long interspersed nuclear element
LTR: Long terminal repeat
MITE: Miniature inverted-repeat transposable element
PBS: Primer binding site
RT: Reverse stranscriptase
SINE: Short interspersed nuclear element
TE: Transposable element
TES: Transcription end site
TREP: Transposable element platform
TSS: Transcription start site
