# Supplementary material for: The repetitive landscape of the 5100 Mbp barley genome
Source: Mob DNA. 2017 Dec 20;8:22. doi: 10.1186/s13100-017-0102-3 (PMC5738225; doi:10.1186/s13100-017-0102-3)
Supplement: Additional file 1: — Supplementary Tables and Figures. (PDF 3776 kb) [file 13100_2017_102_MOESM1_ESM.pdf]

Additional file 1

Supplementary Tables and Figures

Table S1. Numbers of retrotransposons that were found within 10 kb of the transcription start and end sites (TSS and TES) of genes.

Retrotransposons within 10 kb upstream of the TSS of genes

Superfamily	Total	Forward	Reverse	Forward[%]	Reverse[%]
RIX (non-LTR *LINE*)	3672	1895	1777	51.6	48.4
RSX (non-LTR *SINE*)	470	228	242	48.5	51.5
RLX (LTR unknown)	92804	46179	46625	49.8	50.2
RLG (LTR *Gypsy*)	38338	19143	19195	49.9	50.1
RLC (LTR *Copia*)	43862	21686	22176	49.4	50.6
**Total**	**179146**	**89131**	**90015**	**49.7**	**50.2**

Retrotransposons within 10 kb downstream of the TES of genes

Superfamily	Total	Forward	Reverse	Forward[%]	Reverse[%]
RIX (non-LTR *LINE*)	4108	1961	2147	47.7	52.3 [a]
RSX (non-LTR *SINE*)	591	276	315	46.7	53.3
RLX (LTR unknown)	86954	43393	43561	49.9	50.1
RLG (LTR *Gypsy*)	35460	17435	18025	49.2	50.8
RLC (LTR *Copia*)	42983	21169	21814	49.2	50.8
Total	170096	84234	85862	49.6	50.4

[a]Significant enrichment of TEs in reverse orientation relative to gene and p=0.05.

Table S2. Numbers of retrotransposons that were found within 500 of the transcription start and end sites (TSS and TES) of genes.

Retrotransposons within 500 bp upstream of the TSS of genes

Superfamily	Total	Forward	Reverse	Forward[%]	Reverse[%]
RIX (non-LTR *LINE*)	218	131	87	60.1	39.9 [a]
RSX (non-LTR *SINE*)	30	15	15	50	50
RLX (LTR unknown)	4909	2464	2445	50.2	49.8
RLG (LTR *Gypsy*)	1684	885	799	52.6	47.4
RLC (LTR *Copia*)	1792	889	903	49.6	50.4
Total	8633	4384	4249	50.7	49.3

Retrotransposons within 500 bp downstream of the TES of genes

Superfamily	Total	Forward	Reverse	Forward[%]	Reverse[%]
RIX (non-LTR *LINE*)	366	188	178	51.4	48.6
RSX (non-LTR *SINE*)	54	33	21	61.1	38.9
RLX (LTR unknown)	4302	2143	2159	49.8	50.2
RLG (LTR *Gypsy*)	1455	691	764	47.5	52.5
RLC (LTR *Copia*)	1777	824	953	46.4	53.6[b]
Total	7954	3879	4075	48.8%	51.2

[a]Significant enrichment of TEs in forward orientation relative to gene and p=0.05.

[b]Significant enrichment of TEs in reverse orientation relative to gene and p=0.05.

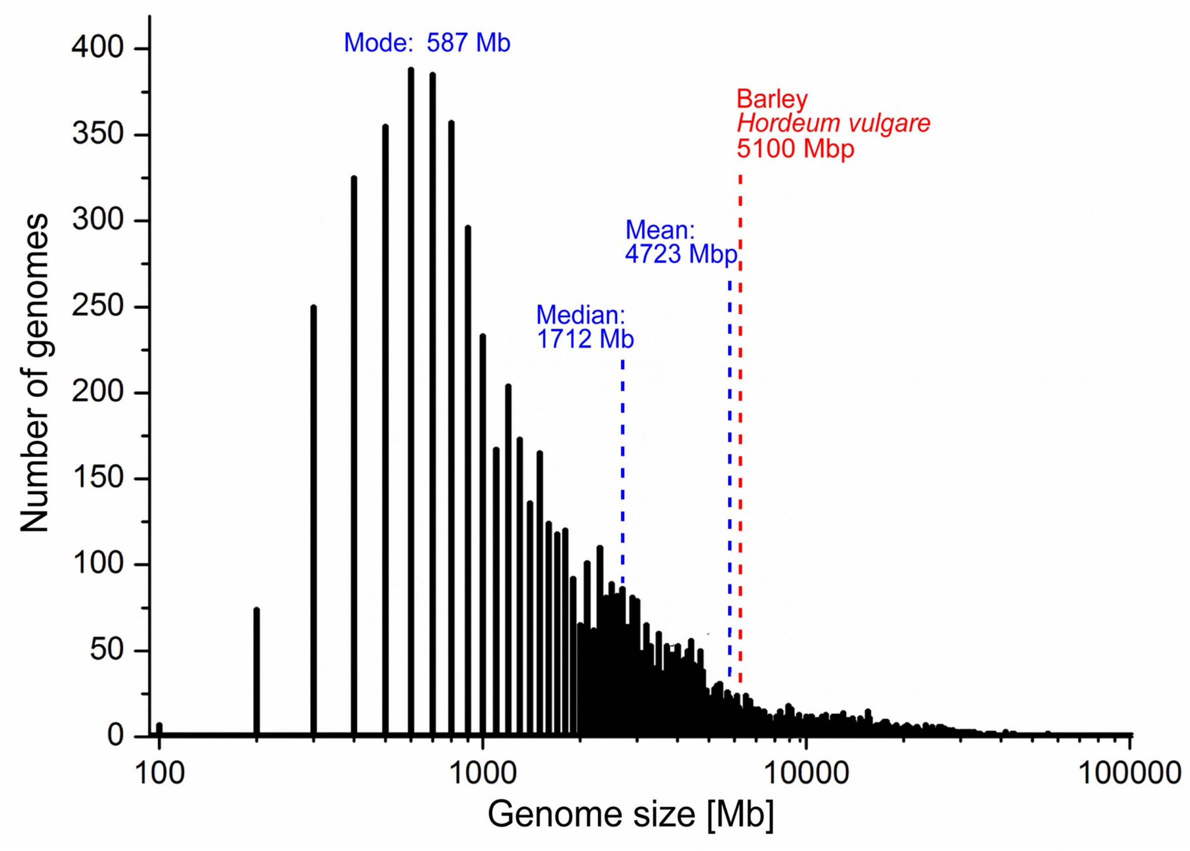

Figure S1. Distribution of 7,541 measured angiosperm monoploid genome sizes (Data source: data.kew.org/cvalues , accessed 07. Feb. 2017). Genome size in bins of 100 Mb is plotted against the number of genomes reported for each size bin. The mean angiosperm monoploid genome is 4,723 Mb, while the median is 1712 Mb. The modal (peak) genome size is 587 Mb. The largest measured genome size is 101,370 Mb, whereas the smallest is 63 Mb. The barley (*H. vulgare*) genome is shown as a red line at 5,600 Mb.

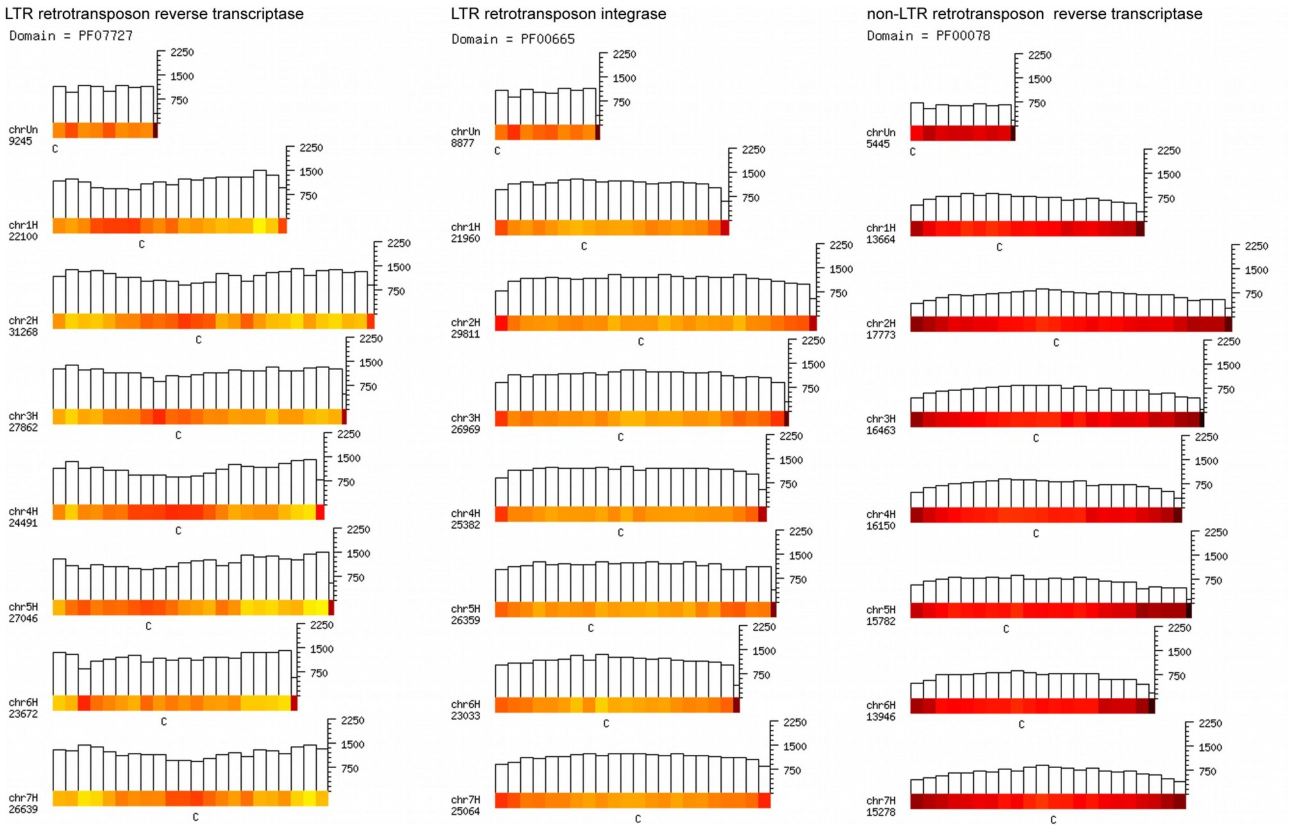

Figure S2. Distribution of annotated integrase and reverse transcriptase domains across the barley genome. The chromosomes were divided into bins of 30 Mb (x-axis). The y-axis indicates the number of respective domains were found in a given bin. The number underneath the chromosome name indicates the total number of integrase or reverse transcriptase protein domains that were annotated on the chromosome. Reverse transcriptase of LTR retrotransposons (PFAM domain PF07727) occur about as frequently as integrase domains, reflecting the fact that both domains are usually present in LTR retrotransposons. Reverse transcriptase of non-LTR retrotransposons (PFAM domain PF00078) are less abundant.

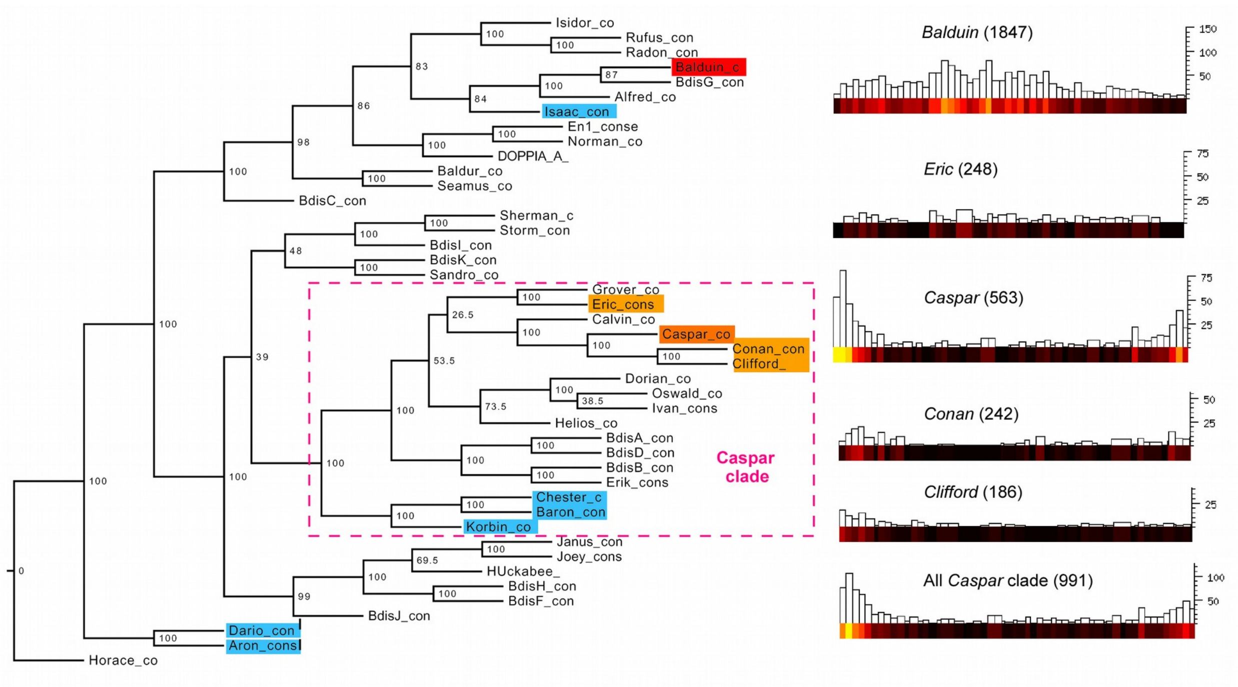

Figure S3. Phylogeny and distribution of *CACTA* elements in the barley genome. For the phylogenetic tree, 44 predicted *CACTA* transposase proteins deposited at TREP were used. *CACTA* sequences come from *Brachypodium*, Sorghum, rice, *Arabidopsis* and Triticeae. High-copy elements from Triticeae are highlighted in orange and red, while Triticeae low-copy families are highlighted in blue. Chromosomal distributions (shown is H1 as a representative) are shown at the right in windows of 10 Mbp. Total copy number on chromosome 1 are given in parentheses next to the family name. *DTC_Balduin* dominates in centromeric regions, while elements of the *DTC_Caspar* clade occupy centromeres. It is not clear whether the preference for different chromosomal regions is evolutionarily conserved, as similar analyses have not been done yet in other grasses. However, it is clear that *DTC_Caspar* and *DTC_Balduin* represent ancient lineages that were present already in the common ancestor of the grasses (Buchmann et al., 2014).

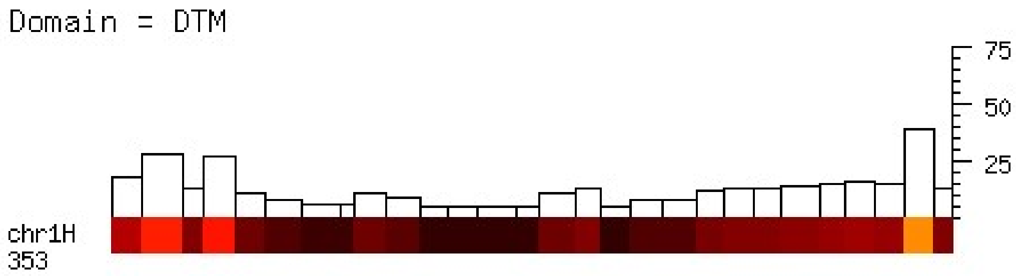

Figure S4. Distribution of *Mutator*-type Transpoase family PFAM domains (PF10551) which some enrichment in distal regions of barley chromosomes.

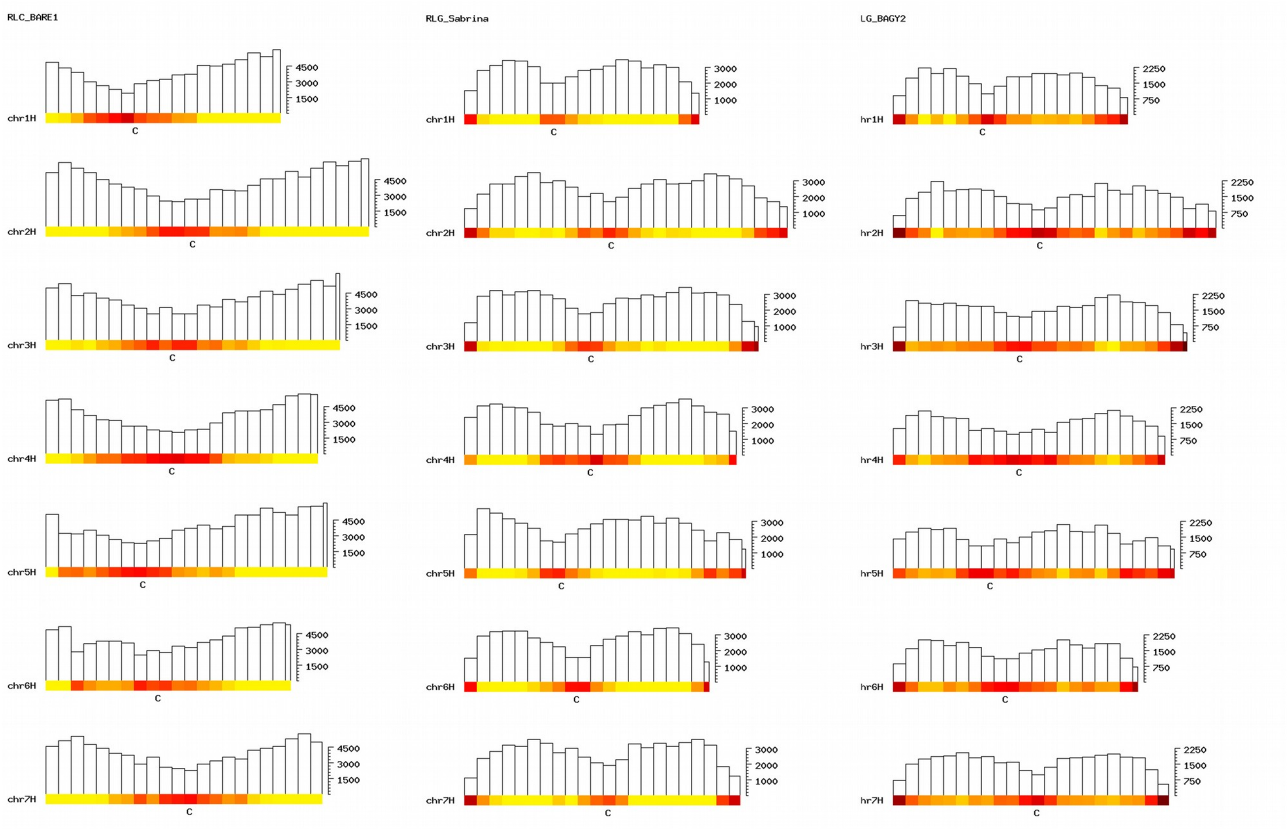

Figure S5. Chromosomal distributions of the three most abundant TE families in the barley genome. Chromosomal distributions are shown at the right in bins of 30 Mb as heat maps and bar plots to indicate absolute numbers. The y-axis indicates the total number of kb that is occupied by the TE family in each bin (Note that scales differ between families).

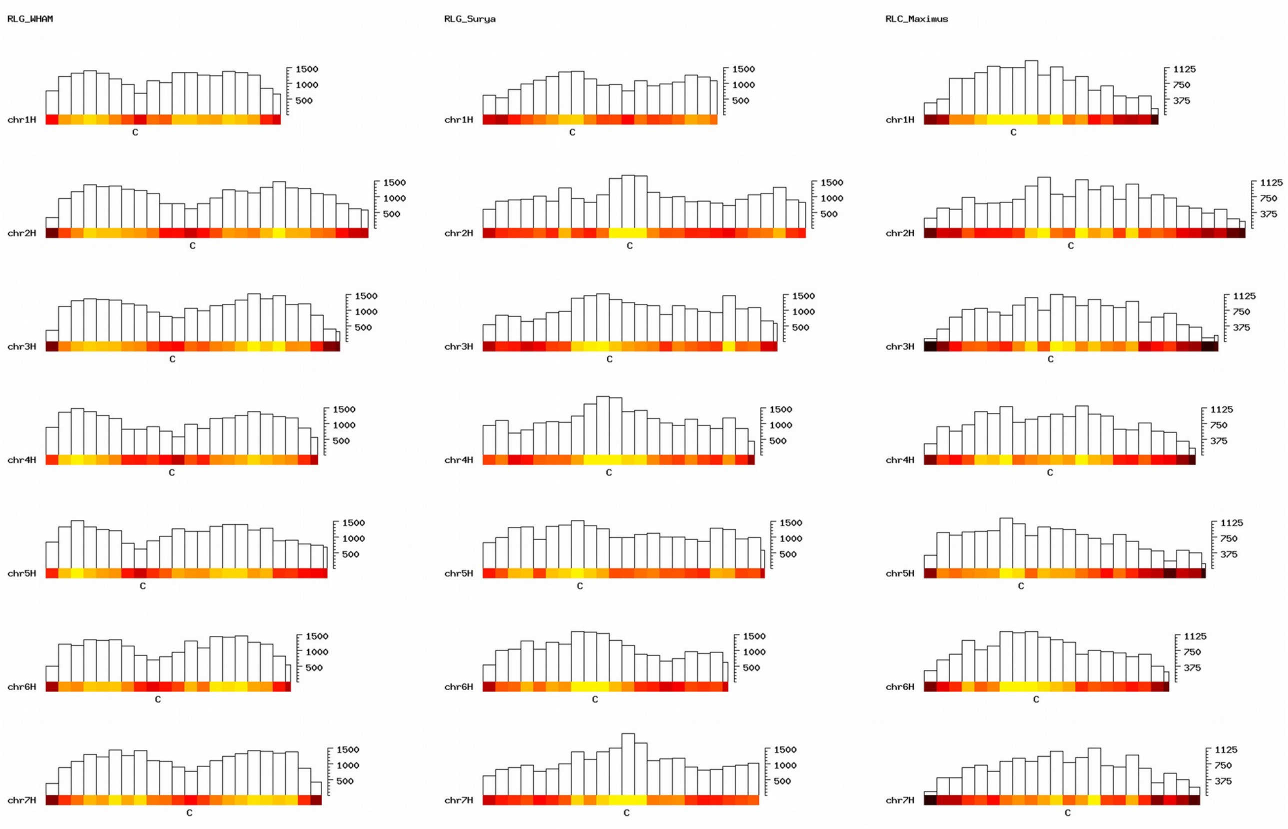

Figure S6. Chromosomal distributions of the 4th to 6th most abundant TE families in the barley genome. Chromosomal distributions are shown at the right in bins of 30 Mb as heat maps and bar plots to indicate absolute numbers. The y-axis indicates the total number of kb that is occupied by the TE family in each bin (Note that scales differ between families).

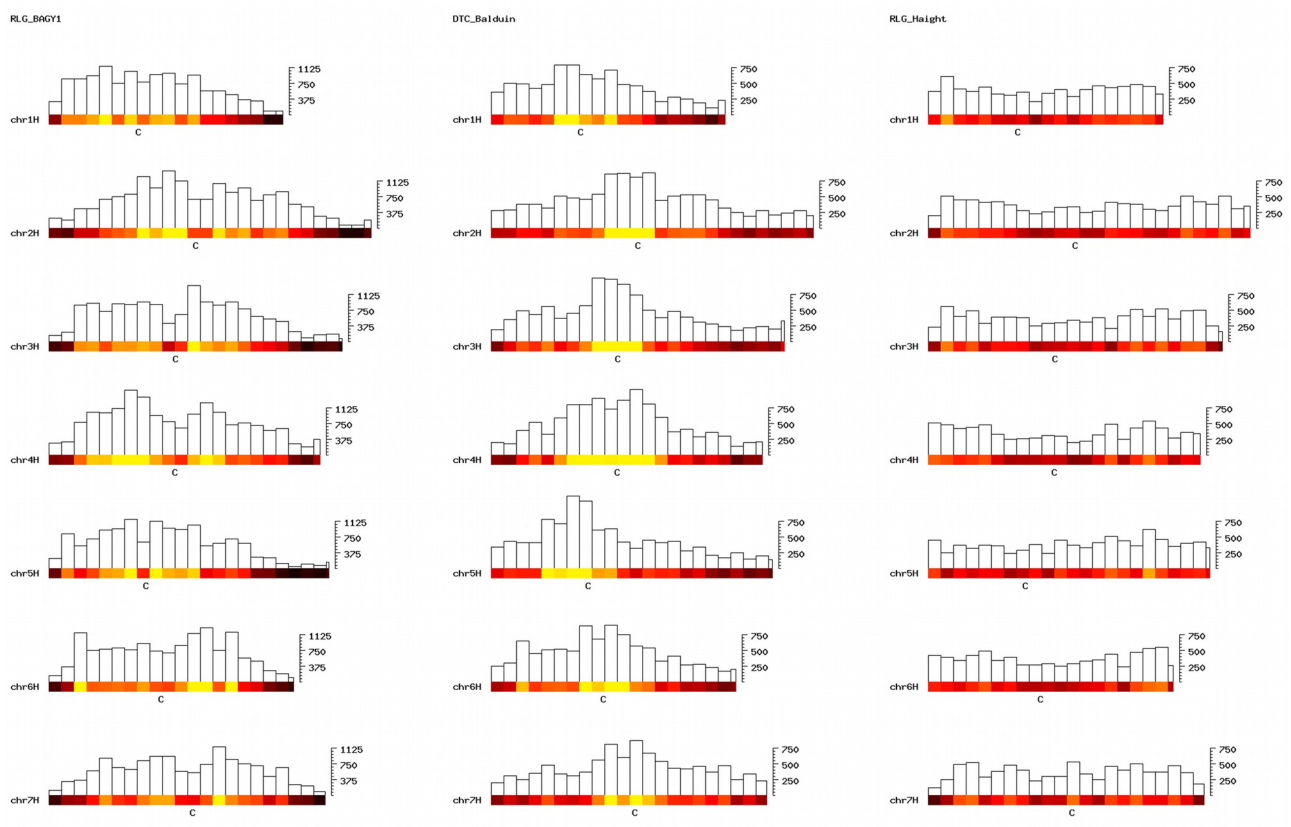

Figure S7. Chromosomal distributions of the 7th to 9th most abundant TE families in the barley genome. Chromosomal distributions are shown at the right in bins of 30 Mb as heat maps and bar plots to indicate absolute numbers. The y-axis indicates the total number of kb that is occupied by the TE family in each bin (Note that scales differ between families).

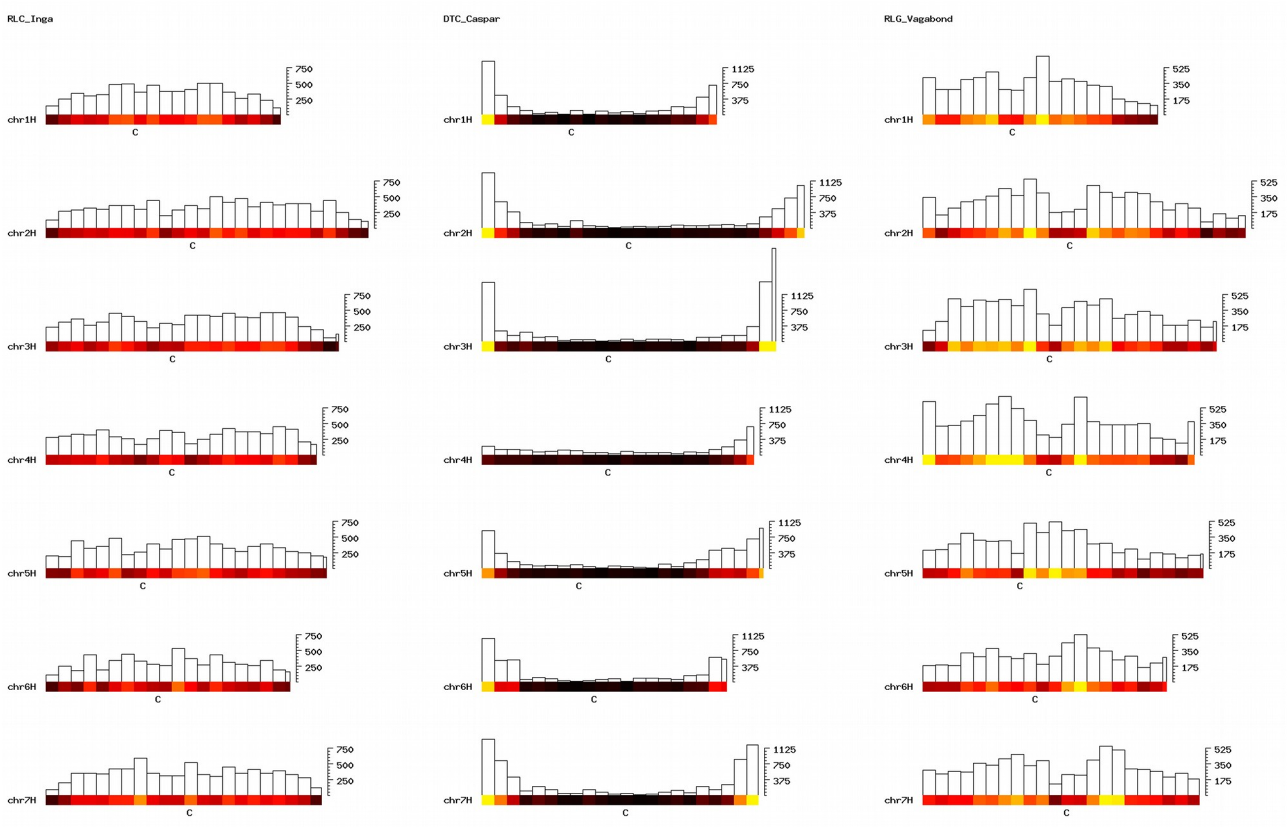

Figure S8. Chromosomal distributions of the 10th to 12th most abundant TE families in the barley genome. Chromosomal distributions are shown at the right in bins of 30 Mb as heat maps and bar plots to indicate absolute numbers. The y-axis indicates the total number of kb that is occupied by the TE family in each bin (Note that scales differ between families).

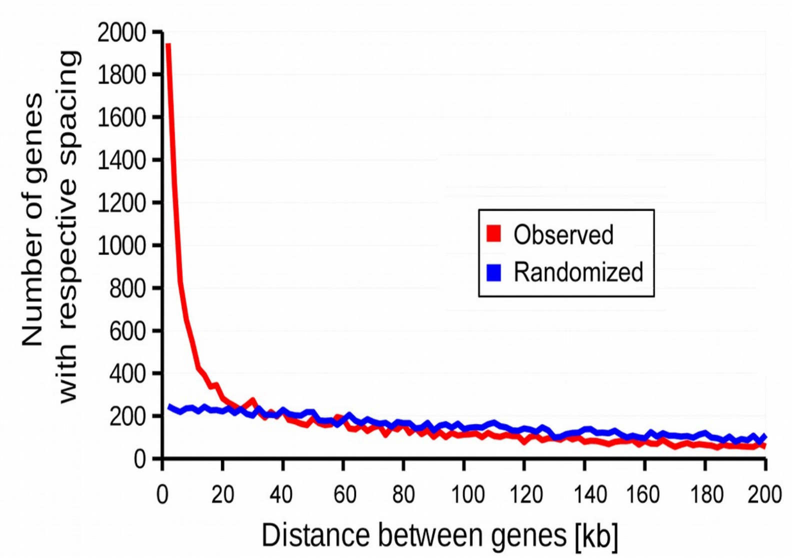

Figure S9. Sizes and numbers of gene clusters in the barley genome. The plot shows frequencies of distances between barely genes. A very large number of genes are found close together (i.e. less than 20 kb). This gene spacing is very different from what would be expected if genes were distributed purely randomly across the genome.

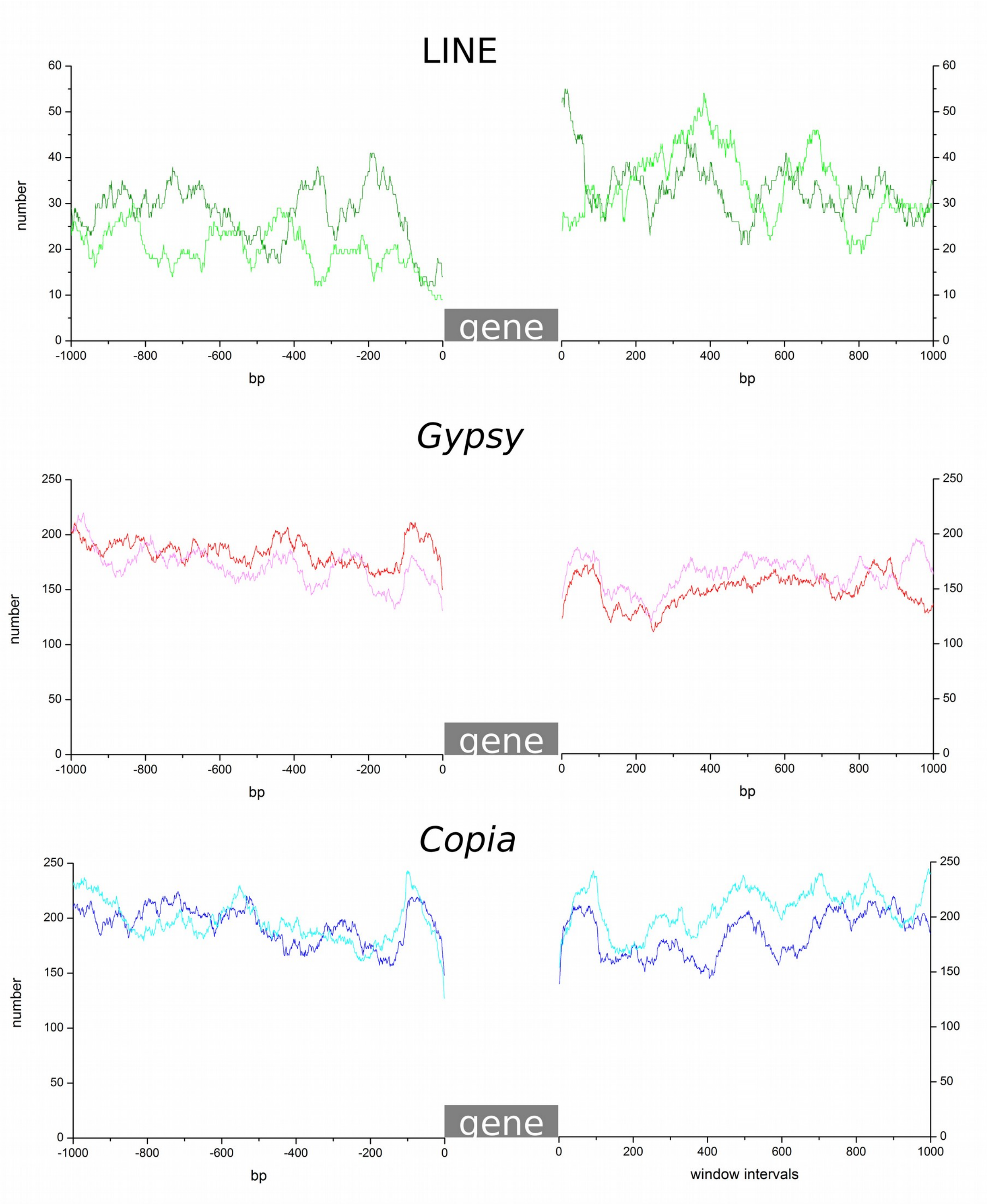

Figure S10. Number of TEs within 1 kb of a high confidence genes in barley. Sense orientation with respect to gene in dark shade, antisense in light shade. *Gypsy* elements appear enriched in sense orientation upstream and antisense downstream. However, the enrichment is not significant based on numbers of identified elements (Table S2).

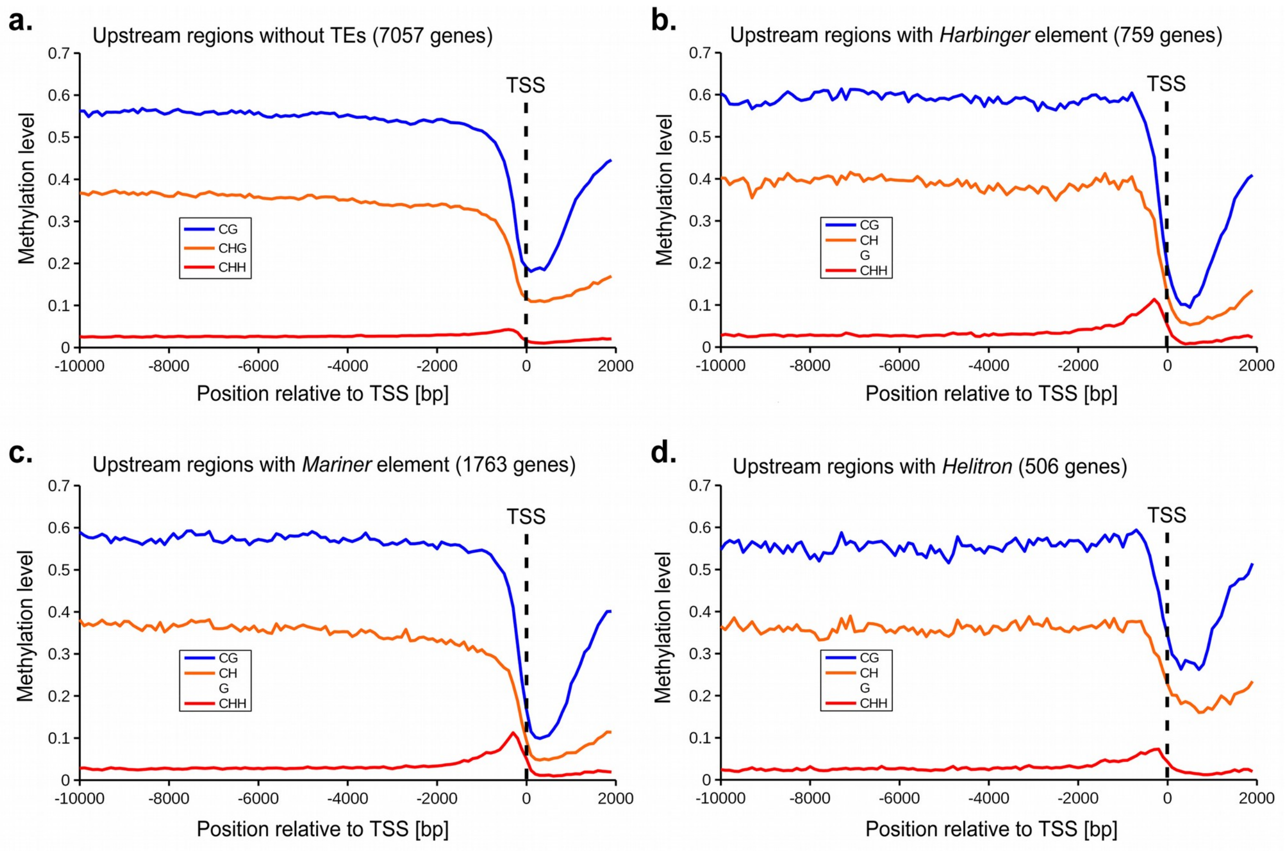

Figure S11. Methylation levels surrounding the predicted transcription start site (TSS) of genes. The graphs show the regions from 10 kb upstream the TSS to 2 kb downstream of it. **a.** Methylation levels in 7,057 genes that do not contain any *Mariner*, *Harbinger* or *Helitron* elements in the region 1.5 kb upstream the TSS to 0.5 kb downstream of it (i.e. the promoter regions). **b.** Methylation levels in 759 genes that contain *Harbinger* elements in the region 1.5 kb upstream the TSS to 0.5 kb downstream of it. **c.** Methylation levels in 1763 genes that contain *Mariner* elements in the region 1.5 kb upstream the TSS to 0.5 kb downstream of it. **b.** Methylation levels in 506 genes that contain *Helitrons* in the region 1.5 kb upstream the TSS to 0.5 kb downstream of it.

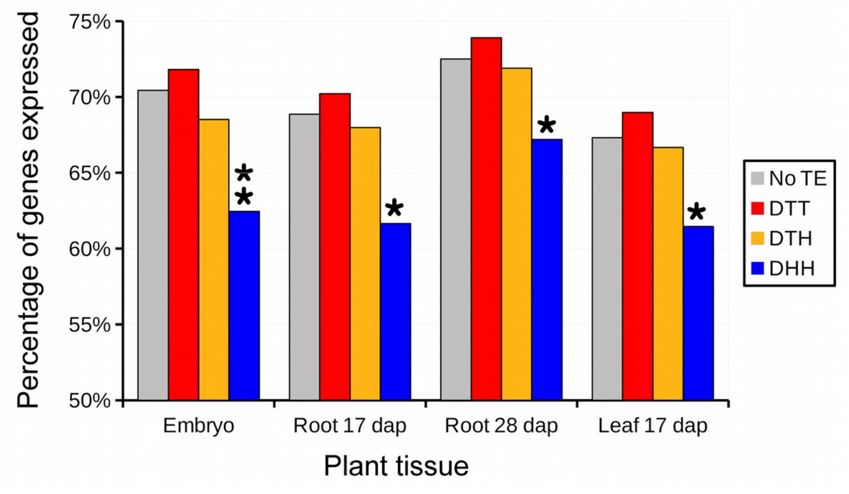

Figure S12. Barley transcriptome data from genes with or without TEs in their upstream regions in four different tissues/developmental stages. Shown are the percentage of genes that show expression (regardless of the level of expression) in the four tissues/developmental stages. Genes with *Helitrons* in their upstream regions are significantly less often expressed than genes without TE in their upstream regions. One asterisk indicates a significant difference at p=0.05, while two asterisks indicates a significant difference at p=0.01.

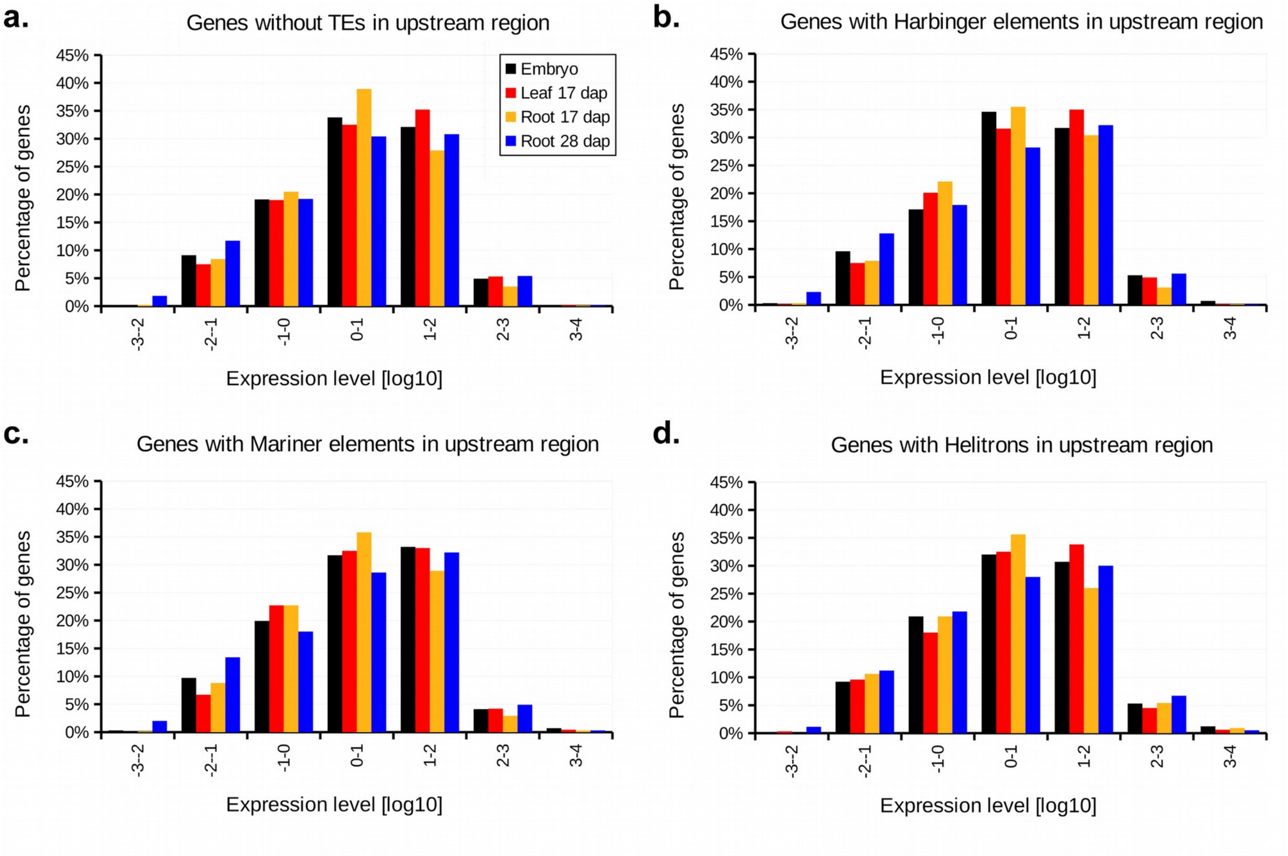

Figure S13. Histograms of transcription levels of genes with or without TEs in their upstream regions in four different tissues/developmental stages. The x-axis indicates the log10 value of the expression levels, while the y-axis indicates the fraction of genes found in the respective class. Because sample sizes differ, the numbers are normalized to fractions of the total sample size. **a.** Expression data from 14,115 genes without *Mariner*, *Harbinger* of *Helitron* transposons in their upstream region; **b.** Expression data from 11,764 genes with *Mariner* transposons in their upstream region; **c.** Expression data from 11,764 genes with *Harbinger* transposons in their upstream region; **d.** Expression data from 11,764 genes with *Helitrons* in their upstream region.

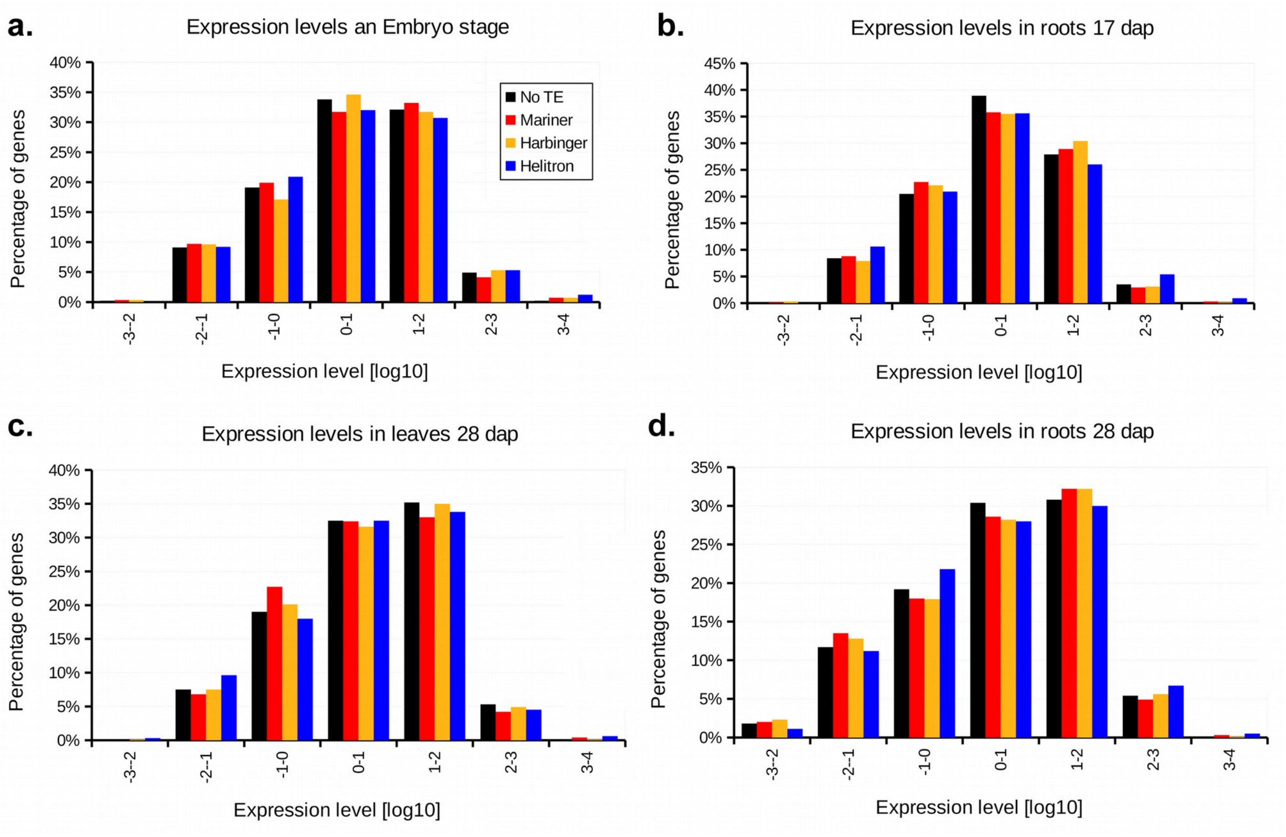

Figure S14. Histograms of transcription levels of genes with or without TEs in their upstream regions in four different tissues/stages. This is the same data as in Figure S14, but ordered according to tissues/developmental stages. The x-axis indicates the log10 value of the expression levels, while the y-axis indicates the fraction of genes found in the respective class. Because sample sizes differ, the numbers are normalized to fractions of the total sample size.

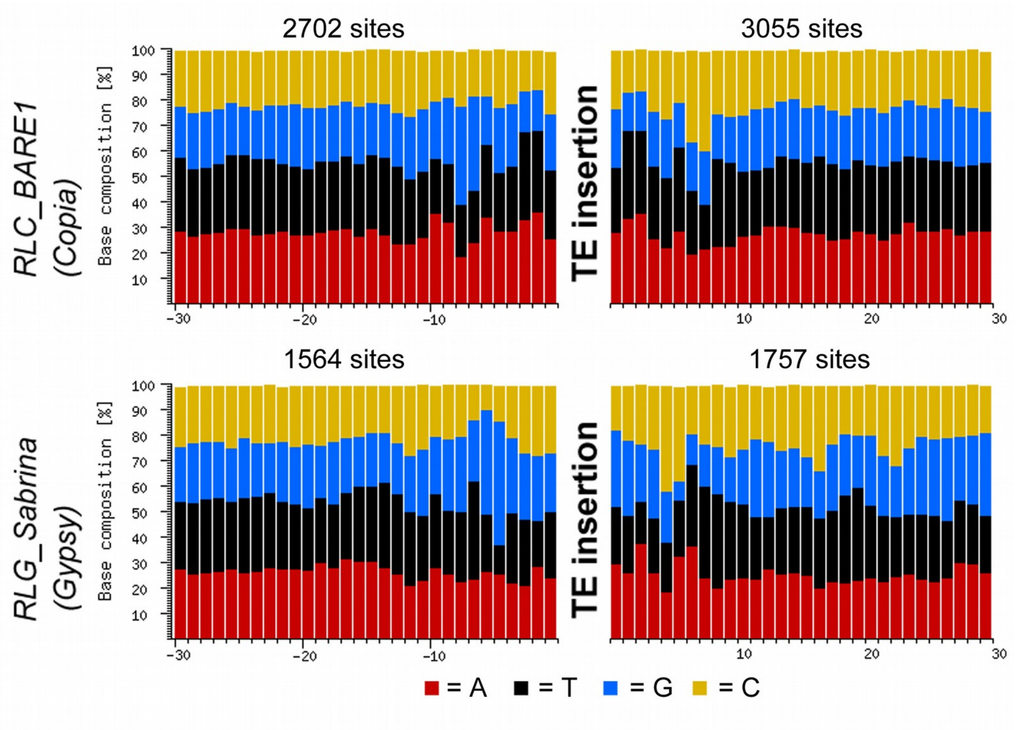

Figure S15. Target site preferences of high-copy LTR retrotransposons from barley. For the plots, the 30 bp flanking intact retrotransposon ends on both sides were collected. Since LTR retrotransposons are often truncated, numbers for 5' and 3' ends are not identical. The different nucleotides at each position were counted across across all insertion sites of a given TE type. The x-axis is the bp position relative to the TE insertion site, while the y-axis shows the relative nucleotide composition for each position.
